# The Novel Methylation Biomarker NPY5R Sensitizes Breast Cancer Cells to Chemotherapy

**DOI:** 10.3389/fcell.2021.798221

**Published:** 2022-01-11

**Authors:** Jiazhou Liu, Xiaoyu Wang, Jiazheng Sun, Yuru Chen, Jie Li, Jing Huang, Huimin Du, Lu Gan, Zhu Qiu, Hongzhong Li, Guosheng Ren, Yuxian Wei

**Affiliations:** ^1^ Chongqing Key Laboratory of Molecular Oncology and Epigenetics, The First Affiliated Hospital of Chongqing Medical University, Chongqing, China; ^2^ Department of Endocrine and Breast Surgery, The First Affiliated Hospital of Chongqing Medical University, Chongqing, China; ^3^ Department of Respiratory, The First Affiliated Hospital of Chongqing Medical University, Chongqing, China; ^4^ Department of Oncology, The First Affiliated Hospital of Chongqing Medical University, Chongqing, China

**Keywords:** breast cancer, NPY5R, WGCNA, IL6, stat3, CpG methylation

## Abstract

Breast cancer (BC) is the most common tumor in women, and the molecular mechanism underlying its pathogenesis remains unclear. In this study, we aimed to investigate gene modules related to the phenotypes of BC, and identify representative candidate biomarkers for clinical prognosis of BC patients. Using weighted gene co-expression network analysis, we here identified NPY5R as a hub gene in BC. We further found that NPY5R was frequently downregulated in BC tissues compared with adjacent tumor-matched control tissues, due to its aberrant promoter CpG methylation which was confirmed by methylation analysis and treatment with demethylation agent. Higher expression of NPY5R was closely associated with better prognosis for BC patients. Gene set enrichment analysis showed that transcriptome signatures concerning apoptosis and cell cycle were critically enriched in specimens with elevated NPY5R. Ectopic expression of NPY5R significantly curbed breast tumor cell growth, induced cell apoptosis and G2/M arrest. Moreover, NPY5R also promoted the sensitivity of BC cells to doxorubicin. Mechanistically, we found that NPY5R restricted STAT3 signaling pathway activation through interacting with IL6, which may be responsible for the antitumor activity of NPY5R. Collectively, our findings indicate that NPY5R functions as a tumor suppressor but was frequently downregulated in BC.

## Introduction

Breast cancer (BC) is one of the most common malignant tumors in women, which seriously affects women’s physical and mental health (Sung et al., 2021). Globally, its morbidity and mortality have long occupied the first place among female malignant tumors. Although treatments based on molecular subtype have achieved striking breakthrough in BC, there are still many patients suffering from recurrence and metastasis, leading to unsatisfactory long-term survival (Desantis et al., 2014). Therefore, a more in-depth study of the etiology and pathogenesis of BC is urgently needed to find more reliable diagnostic and prognostic markers, which may provide a promising targetable therapy for BC.

Neuropeptide Y receptor type 5 (NPY5R) is a G-protein coupled receptor which belongs to the subfamily of neuropeptide Y (NPY) receptors mediating the action of endogenous NPY (Kumar et al., 2016). NPY5R is located on human chromosome 4q31-q32 region, encoding 456 amino acids (Kim and Kim, 2018), it is widely distributed in human brain, mostly in cortex, putamen and caudate nucleus, and can inhibit the activity of adenylate cyclase and the appetite (Dumont et al., 1998; Jacques et al., 1998). Mice lacking the NPY5R gene failed to prefer food odors over pheromones after fasting (Horio and Liberles, 2021). Additionally, it was reported that NPY5R was involved in regulating the proliferation and apoptosis of granulosa cells (Urata et al., 2020). More recently, NPY5R was found to be a molecular marker for tumorigenesis of HR (+)/HER2 (−) BC in adolescents and young adults (Yi and Zhou, 2020). However, the function of NPY5R remains poorly characterized and its pathological significance needs to be investigated.

In this study, we harnessed Gene Expression Profiling Interactive Analysis (GEPIA), Gene Expression Omnibus (GEO) and The Cancer Genome Atlas (TCGA) databases to analyze the expression of NPY5R in BC and normal breast tissues and elucidate the relationship between NPY5R expression and prognosis of BC patients. Interestingly, we found that NPY5R expression was silenced by promoter methylation in BC. Our study sheds light on the critical role of NPY5R in inhibiting BC cell growth and increasing the sensitivity of BC cells to doxorubicin (DOX) *in vitro*. Further mechanistic studies showed that the IL6-STAT3 pathway was implicated in NPY5R-mediated antitumor effects. Thus, NPY5R may serve as a biomarker for BC diagnosis and a potential target for BC treatment.

## Materials and Methods

### Data and Resources

The clinical characteristics and RNA-seq data included 113 normal samples and 1109 tumor samples were retrieved from the Breast Invasive Carcinoma (BRCA) project of TCGA database (https://portal.gdc.cancer.gov/). The microarray datasets were collected from the GEO database (https://www.ncbi.nlm.nih.gov/geo/). Datasets TCGA-BRCA and GSE29431 were used to construct the co-expression networks and identify hub genes in this study. The R package of “limma” was used to identify differentially expressed genes (DEGs) according to the criteria of false discovery rate (FDR) < 0.05 and absolute of log2 fold change >1. We explored the expression of NPY5R in BC using GEPIA database (http://gepia.cancer-pku.cn/). Datasets GSE37751 and GSE5364 were utilized to validate the expression of NPY5R in BC.

### Weighted Gene Co-Expression Network Analysis (WGCNA)

Gene co-expression network analysis was performed with the R package “WGCNA” (Langfelder and Horvath, 2008). First, the similarity matrix was constructed by using the expression data by calculating the Pearson correlation coefficient between two genes. Next, the similarity matrix was transformed into an adjacency matrix with a network type of signed. Six and five were set as the soft power using the pickSoftThreshold function in the Datasets TCGA-BRCA and GSE29431, respectively. Then, the gene expression matrix was converted into the Topological Overlap Matrix (TOM). 1-TOM was used as the distance to cluster the genes, and then the dynamic pruning tree was built to identify the modules. Finally, intersection genes of significantly related modules (the blue and brown modules) and DEGs were visualized by Cytoscape software (v3.8.0). The top 10 hub genes, identified by the plug-in cytoHubba of the Cytoscape software with maximal clique centrality (MCC) algorithm.

### Functional Enrichment Analysis

The functional enrichment of NPY5R-coexpressed genes was analyzed by Gene Ontology (GO) classification and Kyoto Encyclopedia of Genes and Genomes (KEGG) pathways from the SHBIO (http://enrich.shbio.com/). Adjust *p* value <0.05 was considered as statistically significant.

### Gene Set Enrichment Analysis (GSEA)

GSEA 4.1.0 software (downloaded from http://www.gsea-msigdb.org/gsea/downloads.jsp) was employed to dissect the signaling pathways significantly associated with NPY5R expression levels. The pathways with the following criteria were regarded to be significantly enriched: nominal *p*-value < 0.05, false discovery rate (FDR) *q*-value < 0.25, and normalized enrichment score (NES) > 1 (Liu et al., 2021).

### Cell Lines and Tissue Specimens

The human BC cell lines MCF-7, BT-549, BT-474, MDA-MB-231, MDA-MB-468, T47D, ZR-75-1, and SK-BR-3, the normal mammary epithelial cell line MCF-10A were obtained from American Type Culture Collection (ATCC, Manassas, VA, United States). Cell lines were cultured in RPMI-1640 medium (Gibco BRL, Karlsruhe, Germany) containing 10% fetal bovine serum at 37°C with 5% CO_2_. Human BC samples and matched adjacent non-tumor tissue samples used for immunohistochemistry (IHC) staining and quantitative real-time PCR (qRT-PCR) were collected from the First Affiliated Hospital of Chongqing Medical University. This research was authorized by the Institutional Ethics Committees of the First Affiliated Hospital of Chongqing Medical University.

### 5-Aza-2-deoxycytidine (Aza) and Trichostatin A (TSA) Treatment

Cells were treated with 10 μM Aza (Sigma-Aldrich, St. Louis, MO, United States) for 72 h and then treated with 100 nM TSA (Cayman Chemical Co., Ann Arbor, MI, United States) for an additional 24 h.

### Cell Proliferation, Clonogenic Assay, Cell Cycle Analysis, and Apoptosis Assay

These experiments were performed as described previously (Li X. et al., 2018; He et al., 2020). DOX (HANHUI, 19052011) was applied at various concentrations (4, 8, 12, and 16 μM) for 48 h and DMSO was used as control (Yang et al., 2021).

### Western Blotting

Western blot analysis was performed as described previously (Li Y. et al., 2018). Western blot analysis was performed with anti-cleaved PARP (#5625), anti-cleaved-caspase9 (#9501), anti-*β*-Tubulin (#2146), anti-p-STAT3 (#9145), and anti-STAT3 (#9139) antibodies purchased from Cell Signaling Technology (Danvers, MA, United States). An anti-NPY5R (#PA5-106850) antibody was purchased from Invitrogen (Waltham, MA, United States). Anti- cyclin B1 (sc-245) antibody was obtained from Santa Cruz Biotechnology., Inc. (Santa Cruz, CA, United States). Anti-cdc25c (#A5133) antibody was purchased from Bimake (Houston, TX, United States).

### qRT-PCR

Total RNA was extracted from cells using the TRIzol extraction kit (Invitrogen). qRT-PCR was carried out as described previously (Li Y. et al., 2018). All the primers used for qRT-PCR were listed in Additional file: Supplementary Table S1.

### Immunofluorescence Assay

Immunofluorescence staining was performed as described in our previous study (Li Y. et al., 2018). The cells were incubated with anti-STAT3 (1:800, CST, #9139). After incubating with secondary antibody, DNA was counterstained with diamidino phenylindole (DAPI, Sigma–Aldrich, 32670). Imagines were obtained under a confocal laser scanning microscope (Leica, Hilden, Germany).

### Immunohistochemistry

The IHC protocol was performed as described previously (Li Y. et al., 2018). IHC staining was performed from human BC and adjacent tissues using anti-NPY5R (1:50, Invitrogen, #PA5-106850). Based on the immunoreactive score method, the intensity of human BC tissue staining (protein expression) was scored range from 0-10, indicating negative staining to strong staining.

### Plasmids and Cell Transfection

A NPY5R-expressing plasmid was purchased from GeneChem (Shanghai, China). pcDNA3.1 and NPY5R-containing plasmid (4 μg) were transfected with Lipofectamine 2000 (Invitrogen, Carlsbad, United States) into MDA-MB-231 and SK-BR-3 cells. After 6 h of transfection, cells were changed fresh cell culture medium for subsequent experiments.

### Random Forest Screening for Important Genes

Random forest algorithm was leveraged to evaluate the impact of the expression of select genes on patient OS with the R package “randomForest”, and to provide variable importance value for each gene (Tian et al., 2020).

### Statistical Analysis

All experiments were repeated three times. Data analysis was performed by GraphPad Prism 7 and in the R version 4.1.0. We used the *t*-test and Wilcox test to calculate the difference between high-NPY5R and low-NPY5R groups. Survival curves were calculated using the Kaplan-Meier method and were compared with the log-rank test. (*p < 0.05; **p < 0.01; ***p < 0.001; ****p < 0.0001. *p* values <0.05 were considered statistically significant).

## Results

### Key Gene Module Related to Breast Cancer is identified *via* WCGNA

**FIGURE 1:**
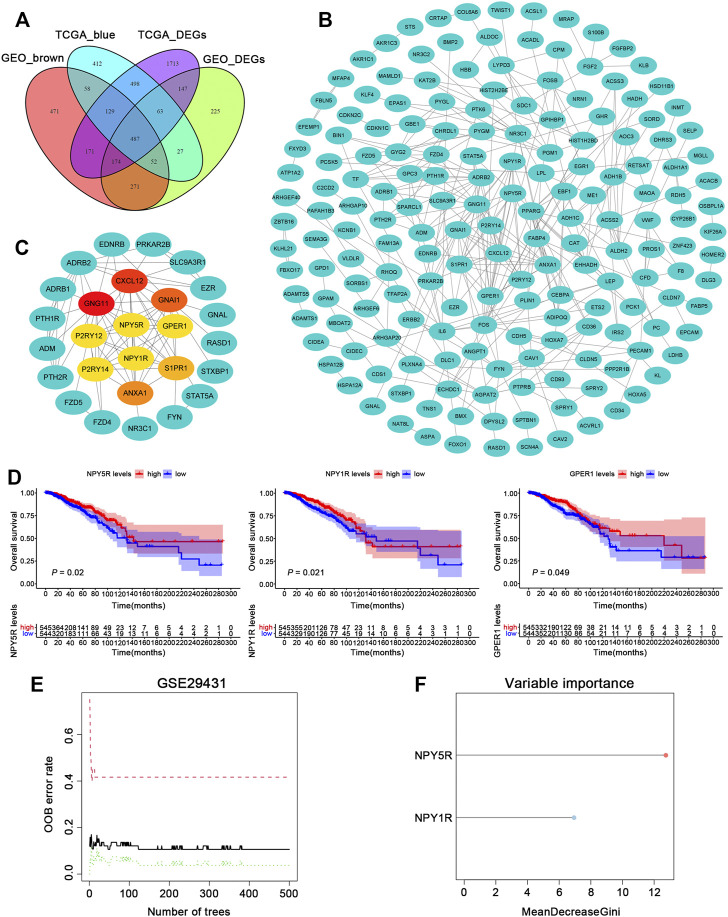
Identification of gene modules during BC development using WGCNA. **(A)** Venn diagram depicting the overlap between DEGs and WGCNA gene modules that are the most relevant to tumorigenesis. **(B)** PPI network of overlapping genes was selected from the Venn diagram. **(C)** According to the MCC score, the Top 10 genes were selected as the hub genes. **(D)** The Kaplan–Meier survival analysis of ten hub genes in TCGA-BRCA cohort. **(E)** The influence of the number of decision trees on the error rate. The *x*-axis is the number of decision trees and the *y*-axis is the OOB error rate. **(F)** Results of the Gini coefficient method in random forest classifier. The gene (red lollipop) that ranked in the top list according to the prognostic importance was chosen for further analyses.

To dig out relevant genes contributing potentially to the pathogenesis of BC, we analyzed the TCGA-BRCA and GSE29431 datasets to examine differentially expressed genes (DEGs). 3381 and 1445 DEGs were extracted from the expression profiles in the two datasets, respectively (Supplementary Figures S1A,B). To further identify BC phenotype-related modules, we performed WGCNA based on gene expression profiles in the above datasets. The co-expression modules were generated with Dynamic tree cutting. 19 and 11 genes co-expression modules were recognized in the TCGA-BRCA and GSE29431 datasets, respectively (Supplementary Figures S1C,E). By performing a module trait relationship analysis, the blue module and the brown module were identified in datasets TCGA-BRCA and GSE29431, respectively, for their significantly negative correlation with tumor development (Supplementary Figures S1D,F). The list of genes in blue and brown modules is provided in Supplementary Table S2. Finally, we obtained intersection genes of blue and brown modules genes and DEGs in datasets TCGA-BRCA and GSE29431 (Figure 1A).

### NPY5R has the Potential to Act as a Regulatory Hub in Breast Cancer

To identify putative novel targets in BC, 487 overlapped genes were selected for further analysis. Based on the STRING database (https://www.string-db.org/), we built and presented a PPI network through Cytoscape app, and then calculated the hub genes of the protein network using the cytoHubba plugin (Figure 1B). As shown in Figure 1C, we obtained the top 10 hub genes (CXCL12, GNG11, GNAI1, P2RY12, P2RY14, ANXA1, S1PR1, NPY5R, NPY1R, and GPER1) by MCC values. To investigate the relationship between the 10 hub genes and patient prognosis, the patients in TCGA-BRCA cohort were assigned to groups based on high or low expression of the 10 genes. The expression of 3 hub genes (NPY5R, NPY1R and GPER1) was found closely correlated with BC patients’ overall survival (OS) as determined by K–M analysis, shown in Figure 1D (*p* < 0.05, log-rank test). In the GSE29431 dataset, a machine learning algorithm called random forest (RF) classifier identified a model combining two genes (NPY5R, NPY1R; Figure 1E). One gene (NPY5R) that has rarely been reported among most cancers ranked at the top of the list according to the prognostic importance (Figure 1F). Therefore, NPY5R was selected for in-depth investigation.

### NPY5R is Downregulated in Human Breast Cancer

**FIGURE 2:**
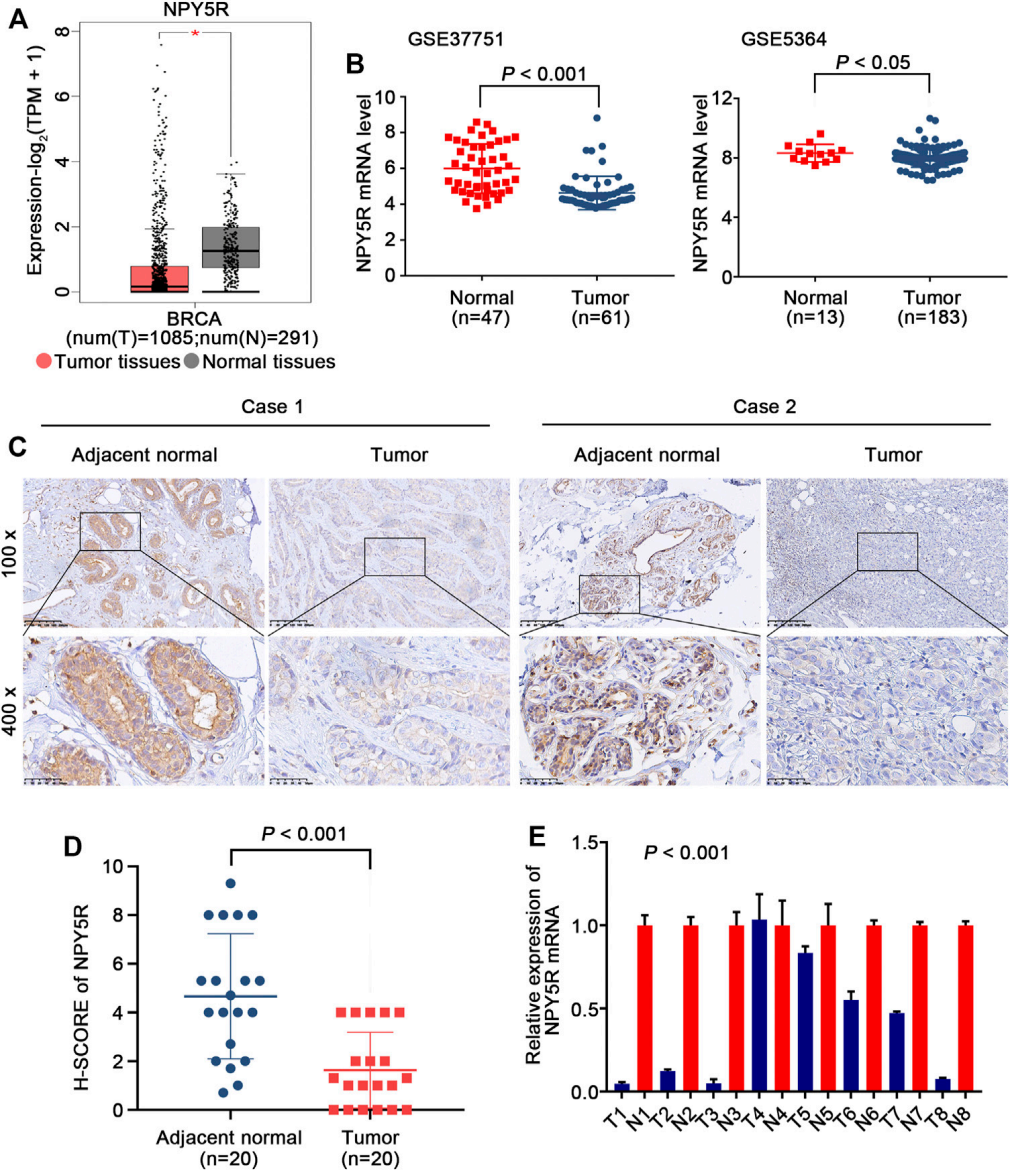
The expression levels of NPY5R in BC tissues. **(A)** NPY5R mRNA expression in BC and normal breast tissues from TCGA database (*p < 0.05). **(B)** Analysis of NPY5R expression in BC and normal breast tissues using the GEO database. Statistical significance was evaluated using Wilcoxon rank sum test. **(C)** Immunohistochemical staining of NPY5R in BC tissues and adjacent non-tumor tissues. Typical images are shown at 200× and 400× magnifications. Scale bars, 50 μm. **(D)** H-SCORE of the two groups (*p* < 0.001). **(E)** 8 pairs of primary BRCA and adjacent tissues are tested by q-PCR (*p* < 0.001).

To explore the significance of NPY5R in BC, we first analyzed publicly available TCGA-BRCA dataset. A significantly decreased NPY5R expression was observed in tumor tissues compared with tumor-free tissues (Figure 2A). As predicted, same trend was demonstrated in two independent datasets (GSE37751, GSE5364) from GEO (Figure 2B). IHC further confirmed that NPY5R protein levels in BC tissues were lower than those in adjacent non-tumor tissue (Figures 2C,D). Furthermore, we also showed that all (7/8 cases) of BC tissues (tumor) exhibited lower NPY5R expression compared to their corresponding non-cancerous controls (normal) by qPCR (Figure 2E).

### Promoter Hypermethylation Contributes to the Decreased Expression of NPY5R in Breast Cancer

**FIGURE 3:**
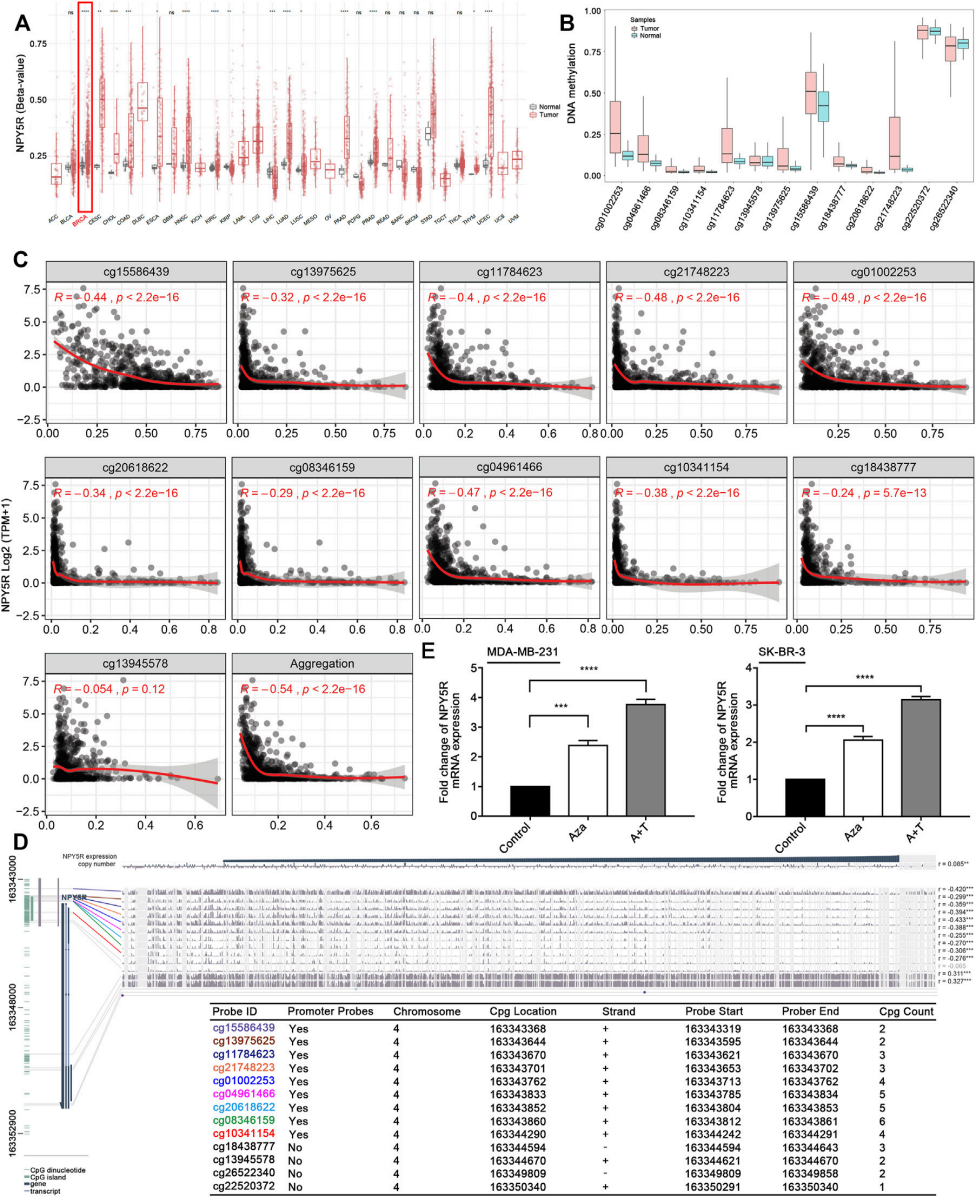
Methylation of NPY5R in BRCA. **(A)** The methylation *β* values of NPY5R in various types of tumoral and their paracancerous tissues in TCGA datasets. **(B)** The methylation level of different probes in breast tumor and normal groups. **(C)** Correlation between NPY5R expression and methylated sites. Spearman’s correlation coefficient (r) was used for the significance test. **(D)** The left aquamarine column represents the sequence of NPY5R, and each probe is marked in the sequence. The statistics on the right hand side show how NPY5R expression and promoter DNA methylation are negatively correlated (Pearson correlation coefficient). The relative position of each probe in NPY5R gene is indicated in the bottom panel. **(E)** Detection of NPY5R expression by RT-PCR after Aza treatment without or with TSA (T) in MDA-MB-231 and SK-BR-3 cells. Data represent the mean ± SD of three independent experiments; *p < 0.05; **p < 0.01; ***p < 0.001; ****p < 0.0001.

To explore the possible mechanism leading to the low expression of NPY5R in BC tissues, we studied the correlation between NPY5R methylation state and its expression levels. Based on SMART (Shiny Methylation Analysis Resource Tool) App (http://www.bioinfo-zs.com/smartapp/), we found that the NPY5R methylation level is significantly higher in BC tissues compared to normal breast tissues (Figure 3A). Furthermore, SurvivalMeth (http://bio-bigdata.hrbmu.edu.cn/survivalmeth) was utilized to explore the DNA methylation-related functional elements. There were 13 CpG sites located in promoter and non-promoter region of NPY5R, twelve of which were differential and one was not (Figure 3B). The average value of twelve differential CpG sites was higher in tumor samples (0.29) than in normal samples (0.22, *p* < 0.01). We also found NPY5R mRNA expression was markedly negatively associated with the methylation levels of the cg15586439, cg13975625, cg11784623, cg21748223, cg04961466, cg20618622, cg01002253, cg08346159, cg10341154, and cg18438777 probes (Figure 3C). Given the close relationship between the methylation of NPY5R promoter and NPY5R expression, we suspected that CpG methylation may regulate NPY5R expression. The precise genomic location of DNA methylation is one of the most important factors in the regulatory effect of DNA methylation on gene expression. We explored the available TCGA DNA methylation data at individual CpGs relating to their precise genomic location using the MEXPRESS tool (https://mexpress.be/). As indicated in Figure 3D, we found the methylation of NPY5R distributed in different regions of the gene using 13 probes (the localization of each probe is presented in the figure, and those localized in the promoter region are highlighted in different colors). Based on the above bioinformatic analyses, we next investigated whether DNA methylation affects NPY5R gene expression. Indeed, it was upregulated after treatment with the DNA methyltransferase inhibitor Aza alone or in combination with the HDAC inhibitor TSA (Figure 3E). These results indicated that alterations in the DNA methylation levels could be the underlying mechanisms responsible for the downregulation of NPY5R.

Furthermore, we analyzed the correlations of NPY5R expression and methylation with clinicopathological features (Supplementary Figures S2A–L), and found that decreased NPY5R expression was correlated with lymph node metastasis (N1 vs. N0, *p* = 0.04) and depth of invasion (T4/T2 vs. T1, *p* < 0.05) (Supplementary Figures S2D,F). In addition, tumors with high NPY5R methylation had significantly lymph node metastasis (N3 vs. N1, *p* < 0.05), depth of invasion (T3 vs. T1, *p* < 0.01), and higher TNM stage (III vs. I/II, *p* < 0.05) (Supplementary Figures S2J–L).

### The Biological Function of Co-expression Genes Related to NPY5R in Breast Cancer

**FIGURE 4:**
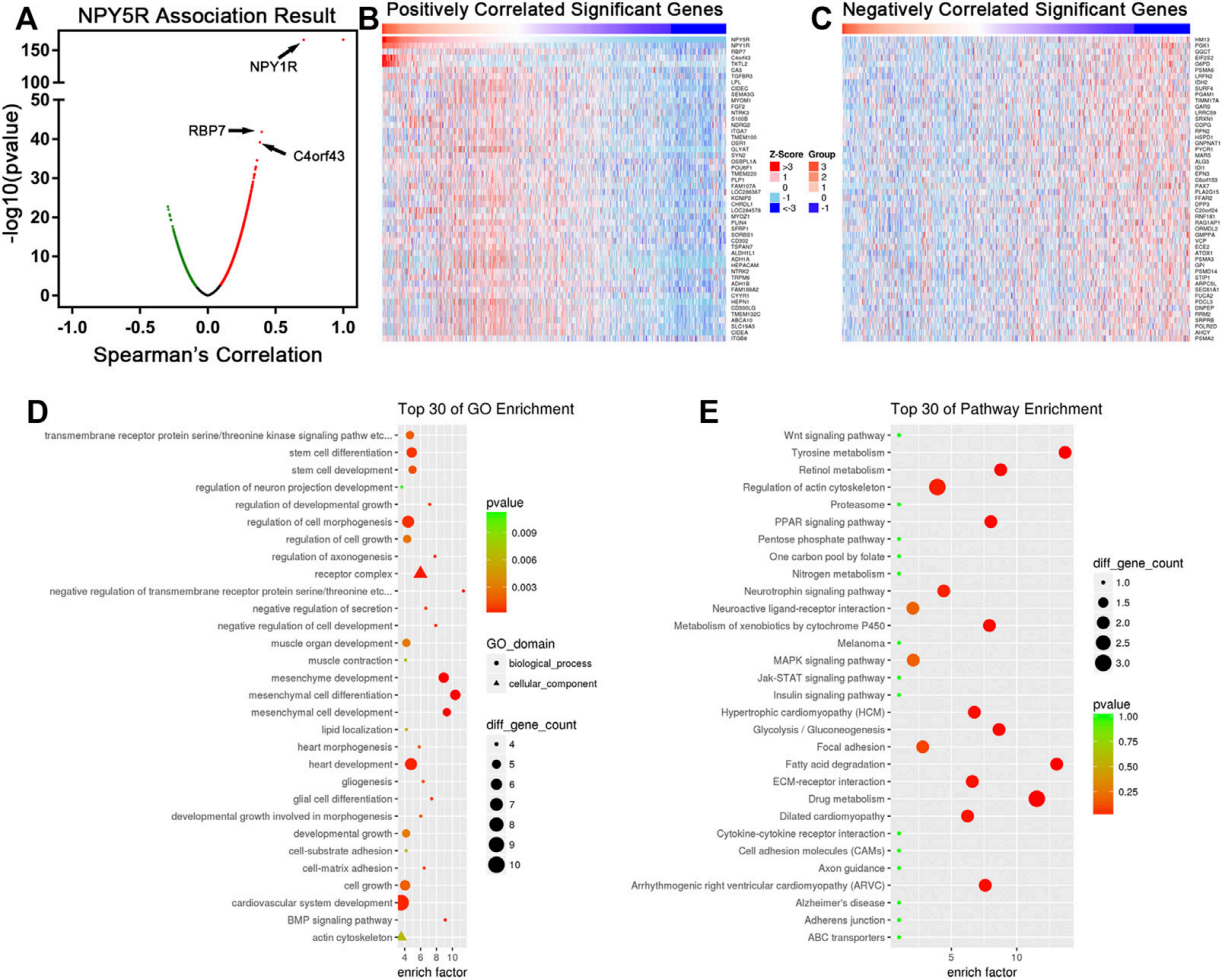
The co-expression genes of NPY5R in BC. **(A)** Strongly co-expressed genes of NPY5R identified by Spearman correlation test in the TCGA BRCA cohort. **(B,C)** Heat maps showing genes positively and negatively correlated with NPY5R in the TCGA BRCA cohort (TOP 50). Red indicates positively correlated genes, and blue indicates negatively correlated genes. **(D,E)** Significantly enriched GO terms and KEGG pathways of NPY5R co-expression genes. FDR, false discovery rate.

To investigate the mechanism of NPY5R regulating BC progression, the co-expression network of NPY5R in the TCGA-BRCA cohort was constructed with functional module of the LinkedOmics database (http://www.linkedomics.org). As shown in the volcano plot (Figure 4A), 5340 genes (dark red dots) were significantly positively correlated with NPY5R, and 2370 genes (dark green dots) were significantly negatively correlated (FDR<0.01, *t*-test followed by multiple testing correction). The top 50 significant genes were presented in heatmaps (Figures 4B,C). Among those, NPY5R was extremely negative associated with HM13 gene expression (negative rank #1, *p* = 2.05e–23). NPY5R also showed strong positive correlations with NPY1R (positive rank #2, *p* = 1.94e–166) and RBP7 (positive rank #3, *p* = 1.42e–42). Furthermore, GO analysis indicated that NPY5R-coexpressed genes were mainly enriched in the developmental growth, cell growth, and stem cell development (Figure 4D), and KEGG analysis demonstrated several enrichment pathways including Jak-STAT, Wnt, and MAPK signaling pathways (Figure 4E).

### NPY5R Suppresses Breast Cancer *via* Inhibiting Cell Proliferation and Inducing Apoptosis and Cell Cycle Arrest

**FIGURE 5:**
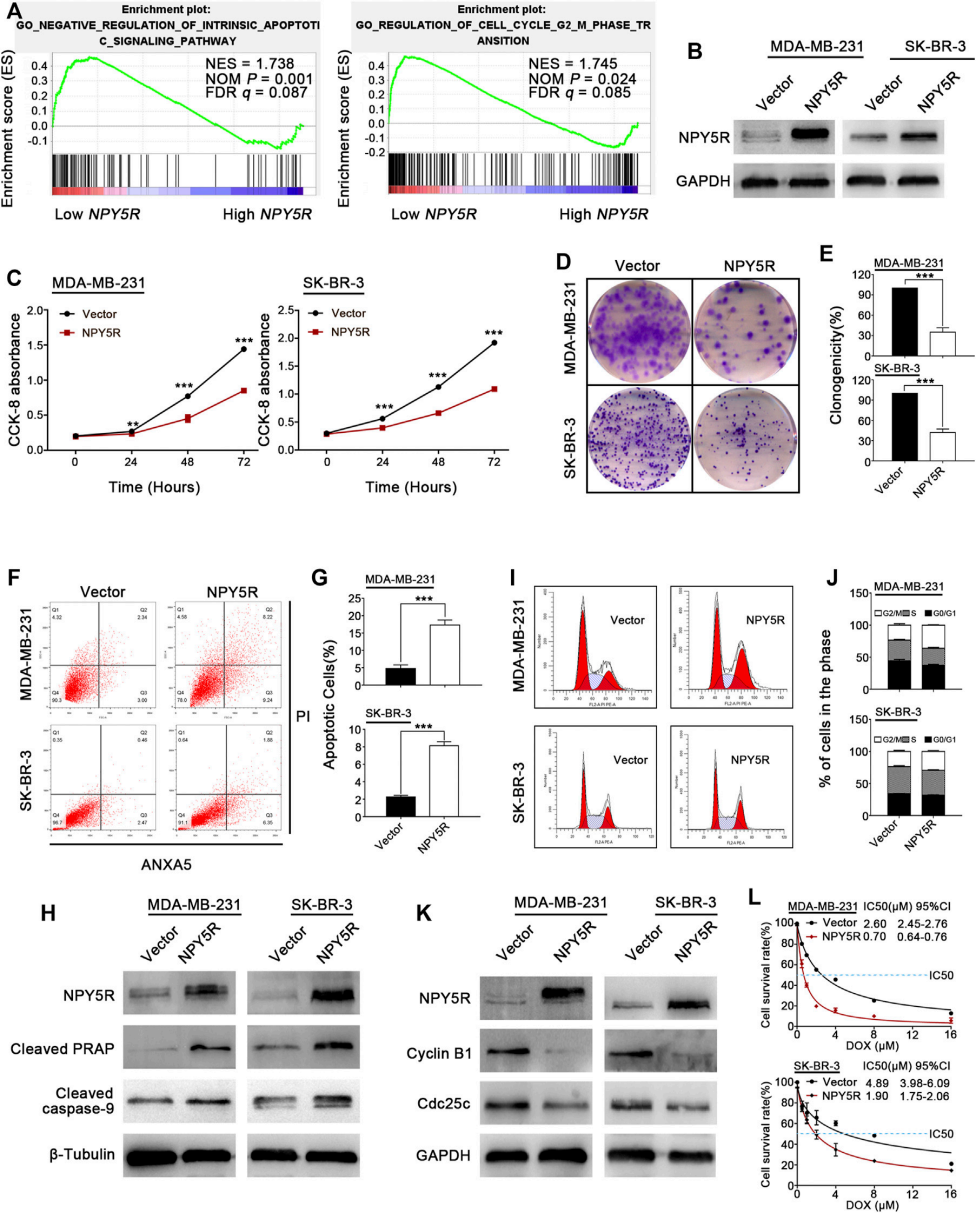
Tumor suppressive functions of NPY5R in BC cells. **(A)** Gene enrichment plots showed that a series of gene sets including GO NEGATIVE REGULATION OF INTRINSIC APOPTOTIC SIGNALING PATHWAY (apoptosis) and GO REGULATION OF CELL CYCLE G2 M PHASE TRANSITION (cell cycle) were enriched in the NPY5R-high subgroup. **(B)** Overexpression of NPY5R in MDA-MB-231 and SK-BR-3 cells were confirmed by western blot. **(C–E)** The effects of transient NPY5R overexpression, the control vector, on cell proliferation and colony formation ability, as measured by CCK-8 **(C)** and colony formation **(D,E)**. Data represent the mean ± SD of three independent experiments; *p < 0.05; **p < 0.01; ***p < 0.001. **(F)** The proportion of apoptotic cells in transiently transfected MDA-MB-231 and SK-BR-3 cells. **(G)** Quantification of apoptosis changes. **(I,J)** Flow cytometry analysis of cell cycle of transiently transfected MDA-MB-231 and SK-BR-3 cells by PI staining. **(H,K)** The expression of apoptosis-related proteins and cell cycle-related proteins in NPY5R-expressing cells was determined by western blot analysis. **(L)** CCK8 was performed to analyze effect of NPY5R expression on chemosensitivity of BC cells to DOX.

To investigate potential biological functions of NPY5R in BC, GSEA was performed with the TCGA-BRCA dataset. The results showed that GO NEGATIVE REGULATION OF INTRINSIC APOPTOTIC SIGNALING PATHWAY (apoptosis) and GO REGULATION OF CELL CYCLE G2 M PHASE TRANSITION (cell cycle) were significantly enriched in the NPY5R-high group (Figure 5A). Next, gain-of-function studies were performed to validate these findings. We transfected pcDNA3.1 (+) framework plasmid or pcDNA-NPY5R plasmid into BC cell lines MDA-MB-231 and SK-BR-3 which lack endogenous NPY5R expression (Supplementary Figure S3). Re-expression of NPY5R mRNA and protein in these cells was evidenced by RT-PCR and western blot analysis (Figure 5B; Supplementary Figure S4). The effects of NPY5R on cell proliferation and viability were further examined *via* CCK8 and colony formation assays. NPY5R overexpression significantly suppressed cell proliferation and viability (Figures 5C–E). To dig out the molecular mechanism by which NPY5R inhibits cell growth, we investigated the effect of NPY5R on cell cycle distribution and apoptosis by flow cytometry. NPY5R increased the proportion of both early and late apoptotic cells in MDA-MB-231 and SK-BR-3 (Figures 5F,G). As cell apoptosis was activated through caspase cascade, the enhanced level of cleaved caspase-9, and poly ADP-ribose polymerase (PARP) was also observed in NPY5R-overexpression cells (Figure 5H). Furthermore, overexpressing NPY5R induced G2/M phase cell cycle arrest, which was confirmed by decreased key G2/M cell cycle regulators, cyclin B1 and cdc25c (Figures 5I–K). To determine the effect of NPY5R expression on the sensitivity of BRCA cells to chemotherapeutic agents, NPY5R-overexpressing cells were treated with DOX at different concentrations for 48 h. Clearly, overexpression of NPY5R enhanced the sensitivity of BC cells to DOX (Figure 5L).

### NPY5R Inhibits IL6-Mediated STAT3 Activation

**FIGURE 6:**
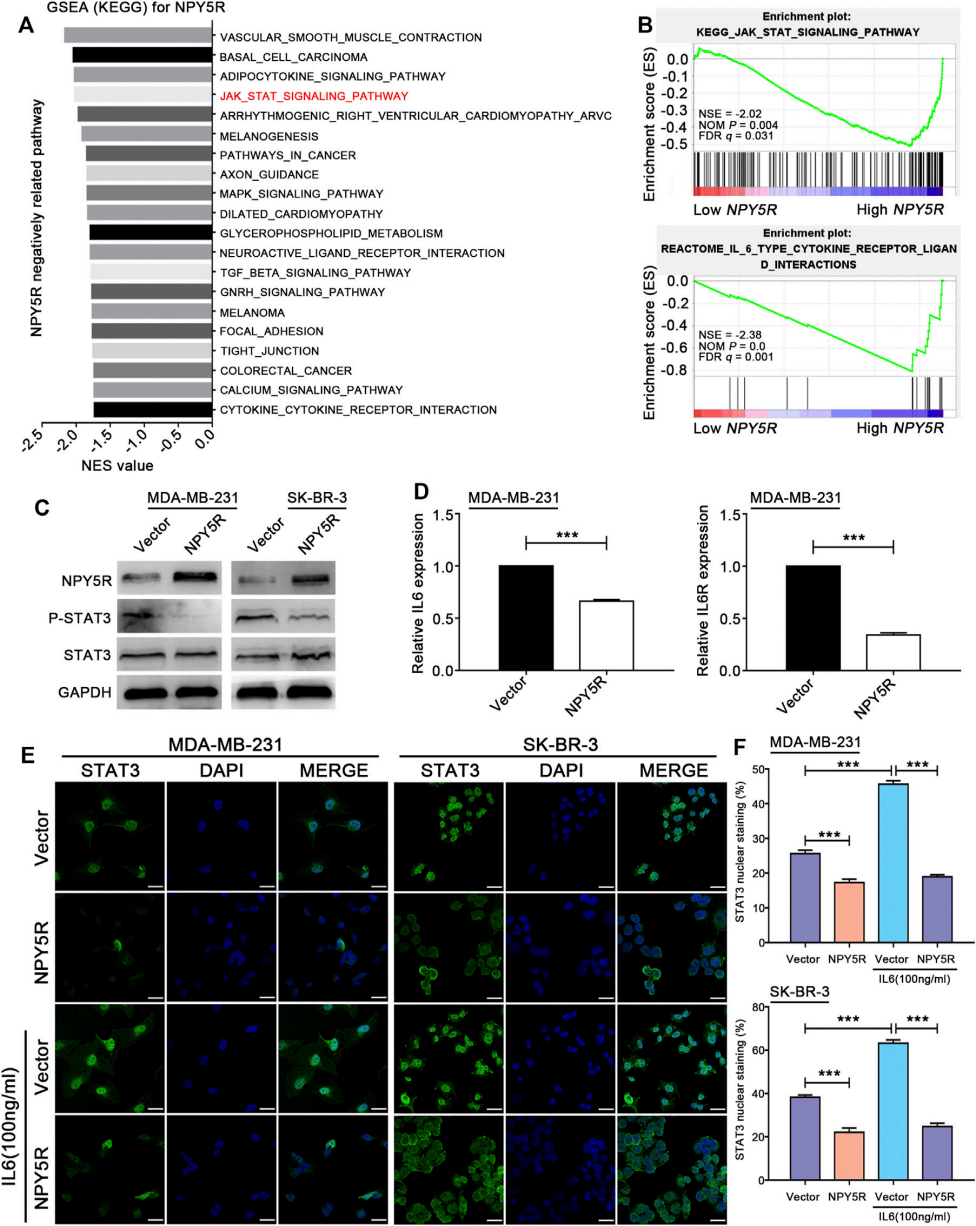
NPY5R antagonizes STAT3 signaling through downregulating IL6. **(A)** GSEA analysis showed NPY5R-related KEGG pathways in BRCA tissue of TCGA database (Low expression group (*n* = 555) vs High expression group (*n* = 554), *p* < 0.05). **(B)** GSEA plots of KEGG JAK-STAT signaling and Reactome IL6 type cytokine receptor ligand interactions showing negatively correlation with higher expression of NPY5R in the BC. NES, normalized enrichment score; NOM, nominal; FDR, false discovery rate. **(C)** The expression of p-STAT3 and STAT3 in NPY5R-expressed MDA-MB-231 and SK-BR-3 cells was detected by western blot analysis. **(D)** The mRNA-expression levels of IL6 and IL6R after NPY5R overexpression in MDA-MB-231 cells. **(E)** Confocal immunofluorescence analysis of STAT3 expression in NPY5R- and empty vector-transfected cells. Cells were cultured and treated with or without IL6 (100 ng ml^−1^). Nuclear localization of STAT3 is shown in green (arrows). DAPI (blue) was used as a nuclear counterstain. Scale bars, 25 μm. **(F)** Quantification of nuclear STAT3-positive staining. Results are presented as means ± S.D.

Next, to investigate which signaling pathways underlie the biological effects of NPY5R, GSEA was performed based on the mRNA expression profiles of NPY5R, and identified 20 gene signatures that were negatively correlated with higher expression of NPY5R. Among them, two established gene signatures KEGG JAK STAT signaling (ranking 4th) and REACTOME IL6 TYPE CYTOKINE RECEPTOR LIGAND INTERACTIONS (ranking 1st) were skewed toward high expression of NPY5R (Figures 6A,B). Therefore, we further examined the effect of NPY5R on these signaling pathways in BC. As shown in Figure 6C, overexpression of NPY5R inhibited the activation of STAT3 signaling. Then, qRT-PCR showed that IL6 and IL6 receptor (IL6R) expressions were downregulated in NPY5R-overexpressed MDA-MB-231 cells (Figure 6D). Since IL6 is a putative activator of STAT3 pathway, we next examined if NPY5R can interfere with IL6-mediated activation of STAT3. Our data indicated that overexpression of NPY5R significantly attenuated the expression of nuclear STAT3. Stimulating MDA-MB-231 and SK-BR-3 cells with IL6 (100 ng ml^−1^) for 6 h led to a significant increase in nuclear localization of STAT3, whereas overexpression of NPY5R significantly abolished this phenomenon (Figure 6E). Taken together, these results showed that IL6-STAT3 presents as an important functional node in mediating the biological effects of NPY5R in BC cells.

## Discussion

Nowadays, the abnormal expression of genes is considered to be one of the factors in occurrence and development of BC, and increasing research has demonstrated that some dysregulated genes in BC might be candidate biomarkers for diagnosis and prognosis. Therefore, we analyzed the datasets TCGA-BRCA and GSE29431 to examine DEGs markedly related to BC pathogenesis. The gene sets (modules) of the WGCNA were constructed from the differential gene expression profiles data *via* using unsupervised clustering, which directly focuses on the relationship between modules and tumorigenesis. Among the modules, we mainly focused on the blue module and the brown module, and the results showed that it was strongly correlated with the tumor phenotype. Using Cytoscape app analysis (Lotia et al., 2013), we obtained the ten most highly connected hub genes (CXCL12, GNG11, GNAI1, P2RY12, P2RY14, ANXA1, S1PR1, NPY5R, NPY1R, and GPER1) in the two modules. Consistent with our results, all these genes have been reported to be related to the development of BRCA (Cooke et al., 2015; Zhu et al., 2017; Shah et al., 2018; Liu et al., 2019; Feng et al., 2020; Zhang H. et al., 2021; Zhang N. et al., 2021; Correia et al., 2021; Xiong et al., 2021). Among them, NPY5R, NPY1R and GPER1 were positively associated with the OS. We further combined the strengths of machine learning techniques to improve the statistical power of our BC predictive model. As a form of an ensemble algorithm, RF has an outstanding performance on the processing of multiple-featured data with high accuracy and precision. RF classifier screening results identified NPY5R as the best-characterized gene. Thus, these data highlight the potential of NPY5R as a clinical prognostic marker in BC.

NPY5R is the major subtype of NPY receptors that mediate the biological functions of NPY (Herzog et al., 1997). NPY and its receptor NPY5R play an essential role in hunger-dependent odour preference (Horio and Liberles, 2021). Notably, the effect of the NPY or NPY receptors on tumor cell growth is controversially discussed (Korner and Reubi, 2007). Y5R agonist had no effect on the growth of this MCF-7 (Memminger et al., 2012). Y5R agonist induced SK-N-MC cell death (Kitlinska et al., 2005). Y2R antagonist prevented the anti-proliferative effects of NPY on cholangiocarcinoma growth (DeMorrow et al., 2011). Overexpressed NPY1R inhibited prostate cancer progression (Li et al., 2020). Moreover, NPY5R in granulosa cells varies among follicular stage and its response is strong at early antral (EA) stage. NPY5R regulates granulosa cell proliferation in a follicular stage-dependent manner, with an induction at EA and suppression at late antral follicles (Urata et al., 2020). Conversely, NPY-induced increases in VEGF expression in 4T1 cells were attenuated only under Y5R antagonism (Medeiros and Jackson, 2013). Y5R antagonist inhibited the proliferative effect of NPY in the 4T1 BC cell line (Medeiros et al., 2012). However, the mechanism of action of NPY5R in the development and progression of BC remains unclear. GEPIA and datasets (GSE37751, GSE5364) were exploited to evaluate the expression of NPY5R in BC tissues compared with normal breast tissues. The results of IHC staining and RT-qPCR further verified that the level of NPY5R was significantly lower in tissues from BC patients than in normal breast tissues. Epigenetic modifications, including DNA methylation, acetylation, etc., can alter gene expression (Jones and Takai, 2001; Cavalli and Heard, 2019). The average *β* values for promoter methylation were significantly higher in the head and neck squamous cell carcinoma (HNSCC) samples than in the normal samples (Misawa et al., 2017). NPY5R promoter methylation correlated inversely with its respective mRNA level in the HNSCC samples (Misawa et al., 2017). Thus, a possible link between promoter methylation and downregulation of NPY5R expression in BC was investigated. Here, we first correlated the expression level of NPY5R and its methylation status. We found an increase in the DNA methylation level of NPY5R in a variety of tumoral samples, and NPY5R mRNA expression was strongly negatively associated with the methylation levels of multiple CpG sites. We further showed that demethylation treatment effectively restored NPY5R expression, confirming that promoter methylation contributes to suppression of NPY5R expression in silenced BC cells. A series of *in vitro* functional experiments revealed that NPY5R possesses a tumor-suppressive function in BC. Although recent report revealed that NPY5R plays a promotive role in the proliferation in high NPY5R-expressing 4T1 cells (Medeiros et al., 2012; Medeiros and Jackson, 2013), our findings indicated that NPY5R overexpression significantly suppressed cell proliferation in MDA-MB-231 and SK-BR-3 cells with the lowest endogenous levels of NPY5R. The divergent effects of NPY5R on tumor cell proliferation may depend on the tumor cell line used, the NPY5R expressed in the cell line, and different experimental conditions such as different NPY5R concentrations.

GSEA suggested that a number of gene sets were found to be negatively correlated with higher expression of NPY5R, including the top-ranking KEGG_VASCULAR_SMOOTH_MUSCLE_CONTRACTION (ranking 1st), KEGG _BASAL_CELL_CARCINOMA (ranking 2nd), KEGG _ADIPOCYTOKINE_SIGNALING_PATHWAY (ranking 3rd), KEGG _JAK_STAT_SIGNALING_PATHWAY (ranking 4th), and REACTOME_IL_6_TYPE_CYTOKINE_RECEPTOR_LIGAND_INTERACTIONS (ranking 1st). The IL6/JAK/STAT3 pathway is aberrantly hyperactivated in many types of cancer (Yu et al., 2014; Johnson et al., 2018), and is important for human BC development as well as BC metastasis (Wang et al., 2017; Siersbaek et al., 2020). Our results demonstrated that re-expression of NPY5R significantly inhibited the phosphorylation and nuclear localization of STAT3. Furthermore, qRT-PCR showed that the expressions of IL6 and IL6R were downregulated in NPY5R-overexpressed cells, indicating a close link between NPY5R and IL6/STAT3.

Together, these data warrant that the potential of NPY5R as a diagnostic and prognostic marker in cancer treatment. Remarkably, the analysis of the methylation levels of NPY5R would help evaluate patient prognosis and efficacy of clinical chemotherapy.

## Data Availability

The original contributions presented in the study are included in the article/Supplementary Material, further inquiries can be directed to the corresponding authors.
