# Supplementary material for: The Novel Methylation Biomarker NPY5R Sensitizes Breast Cancer Cells to Chemotherapy
Source: Front Cell Dev Biol. 2022 Jan 11;9:798221. doi: 10.3389/fcell.2021.798221 (PMC8787223; doi:10.3389/fcell.2021.798221)
Supplement: Supplementary file 1 [file Table1.docx]

**Supplementary Table S1. Sequences of primers used for amplification of target genes**

| Gene primer nucleotide sequence |
| --- |
| NPY5R Forward: 5′-GCTGGATCAGTGGATGTTTGG-3′  Reverse: 5′-CAGATGGCAAAACCTAGTGTCC-3′  IL6 Forward: 5′-ACTCACCTCTTCAGAACGAATTG-3′  Reverse: 5′-CCATCTTTGGAAGGTTCAGGTTG-3′  IL6R Forward: 5′-CATGTGCGTCGCCAGTAGT-3′  Reverse: 5′-AGCTCAAACCGTAGTCTGTAGA-3′  GAPDH Forward: 5′- CAGGAGGCATTGCTGATGAT-3′  Reverse: 5′- GAAGGCTGGGGCTCATTT-3′ |
