# Supplementary material for: The Novel Methylation Biomarker NPY5R Sensitizes Breast Cancer Cells to Chemotherapy
Source: Front Cell Dev Biol. 2022 Jan 11;9:798221. doi: 10.3389/fcell.2021.798221 (PMC8787223; doi:10.3389/fcell.2021.798221)
Supplement: Supplementary file 2 [file Table2.docx]

**Supplementary Table S2. Module genes**

| Blue modules genes | Brown modules genes |
| --- | --- |
| CELF2 | A2M-AS1 |
| IGF2 | AADAC |
| DYSF | AAGAB |
| EFNB2 | AASS |
| GPR34 | ABCA6 |
| EBF2 | ABCA8 |
| LYNX1 | ABCA9 |
| PKIB | ABCC11 |
| RHPN2 | ABCC5 |
| VKORC1L1 | ABCD2 |
| KRT19 | ABHD14B |
| PDGFB | ABHD5 |
| ADSS | ABLIM1 |
| ZSCAN16 | ABLIM3 |
| CANT1 | ACACB |
| TMEM47 | ACADL |
| PCDHB7 | ACBD3 |
| COQ8A | ACER3 |
| DUSP1 | ACKR1 |
| MYO1C | ACO1 |
| EZR | ACSL1 |
| SORBS2 | ACSM5 |
| CNKSR1 | ACSS2 |
| ZNF239 | ACSS3 |
| DQX1 | ACTB |
| SGPL1 | ACTC1 |
| APLNR | ACTRT3 |
| TLN2 | ACVR1C |
| STS | ACVRL1 |
| CEBPA | ADA |
| DLL1 | ADAMTS1 |
| LVRN | ADAMTS2 |
| PROS1 | ADAMTS5 |
| ETFA | ADAMTSL3 |
| PCDH19 | ADAR |
| SSX2IP | ADCK3 |
| TIE1 | ADCY3 |
| TARBP1 | ADCY6 |
| ARHGAP24 | ADH1B |
| ST14 | ADH1C |
| RFTN1 | ADI1 |
| COX7A1 | ADIPOQ |
| TLR4 | ADM |
| LRPPRC | ADRA2A |
| PPL | ADRB1 |
| SEMA5A | ADRB2 |
| KSR1 | ADRBK2 |
| ZNF121 | AF520793 |
| SMIM10 | AFAP1L1 |
| INTS4 | AFTPH |
| NECAB1 | AGPAT2 |
| COL4A2 | AGR2 |
| CDC14B | AGR3 |
| SLC35A2 | AGT |
| JAM2 | AGTR1 |
| SEMA3G | AIF1L |
| PDZRN4 | AIFM2 |
| MARC1 | AK055981 |
| SMARCC1 | AK094644 |
| ZHX3 | AK3 |
| HSPB7 | AKAP1 |
| P3H2 | AKAP12 |
| EPHA2 | AKR1C1 |
| KLB | AKR1C3 |
| RAB3D | ALAD |
| PODXL2 | ALCAM |
| PIGX | ALDH1A1 |
| CAVIN2 | ALDH1L1 |
| FES | ALDH2 |
| SH3RF2 | ALDH3A2 |
| SLCO3A1 | ALDH3B2 |
| EPHA1 | ALDH4A1 |
| MXI1 | ALDH6A1 |
| LCAT | ALDH9A1 |
| GNAZ | ALDOC |
| ADRB1 | ALPK3 |
| AQP1 | AMIGO1 |
| MMP28 | AMOTL2 |
| SUCO | ANGPT1 |
| KLF15 | ANGPTL1 |
| IRAK2 | ANGPTL2 |
| DDIT4L | ANGPTL7 |
| FLVCR1 | ANK3 |
| POLR3GL | ANKDD1A |
| PHYHIP | ANKRD22 |
| SLC12A4 | ANKRD29 |
| MAP6 | ANKRD33B |
| MT-CYB | ANKRD35 |
| CPED1 | ANO3 |
| LRRN2 | ANO5 |
| TESC | ANO6 |
| KLF2 | ANTXR2 |
| EGFL7 | ANXA1 |
| JAZF1 | ANXA11 |
| SPINT2 | ANXA2 |
| IGSF10 | ANXA3 |
| HOXC12 | ANXA9 |
| TMEM100 | AOC3 |
| SULT4A1 | AOX1 |
| PARD3B | AP1M2 |
| RAMP2 | APCDD1 |
| EOGT | APOB |
| SHE | APOD |
| DNAJA4 | APOL6 |
| RDH16 | APOLD1 |
| ME1 | AQP1 |
| NRN1 | AQPEP |
| EDNRB | ARHGAP10 |
| TRAF4 | ARHGAP20 |
| SNX21 | ARHGAP21 |
| ENAH | ARHGAP28 |
| KITLG | ARHGEF19 |
| S100A16 | ARHGEF26-AS1 |
| C11orf96 | ARHGEF37 |
| SAA1 | ARHGEF40 |
| MT-ND5 | ARHGEF6 |
| CTPS2 | ARNT2 |
| PRXL2C | ARRB1 |
| CDC42SE1 | ARSG |
| ACKR1 | ASPA |
| EHBP1 | ASPRV1 |
| EFEMP1 | ASS1 |
| ARHGEF4 | ATF6 |
| STRADB | ATG16L1 |
| CDKN1A | ATOH8 |
| CLMP | ATP1A2 |
| TNN | ATP2B4 |
| B4GALNT3 | ATP2C2 |
| DIAPH2 | ATP6V0A4 |
| SPINT1 | ATP6V0C |
| FBXO41 | ATP6V0E2-AS1 |
| CNNM4 | ATP6V1G1 |
| RNF157 | ATP8B4 |
| LEPR | ATXN1L |
| A2M | ATXN2 |
| HSD17B13 | ATXN7L3B |
| CBLC | AVEN |
| RUSC2 | AX747630 |
| FHOD3 | AX748273 |
| SGK1 | AZIN2 |
| THBS4 | BAALC |
| SGCB | BAIAP2L1 |
| FYN | BANK1 |
| IARS | BC010186 |
| PGAP2 | BC022047 |
| FAM149A | BC062753 |
| PLXND1 | BCAS1 |
| SLC4A4 | BCHE |
| ESAM | BCL2L2 |
| ALDH3A2 | BCL6 |
| VEGFC | BGN |
| PTGER2 | BHMT2 |
| SLC2A3 | BIK |
| AARD | BIN1 |
| FARP1 | BLM |
| STOM | BMP2 |
| RNF122 | BMP5 |
| ARHGAP10 | BMPER |
| EGFR | BMPR1B |
| C3orf14 | BMX |
| NTRK2 | BNC2 |
| TPD52 | BNIP3L |
| CNTNAP2 | BOD1 |
| PIP4K2C | BOK |
| SH2D3C | BPHL |
| NECTIN3 | BPIFB1 |
| MAPK11 | BRE-AS1 |
| MT1M | BRI3 |
| SIK2 | BST1 |
| EBF1 | BTBD6 |
| RPS6KA1 | BTNL9 |
| APCDD1 | BYSL |
| ARHGEF25 | BZW2 |
| DIXDC1 | C10orf10 |
| PAFAH1B3 | C10orf11 |
| GK | C10orf128 |
| SEMA6B | C10orf35 |
| TIPARP | C10orf54 |
| CAV1 | C10orf85 |
| ITGA7 | C11orf70 |
| DHRS3 | C11orf80 |
| PIGM | C11orf96 |
| HIST1H2BG | C14orf180 |
| MEAF6 | C15orf48 |
| MAOB | C16orf59 |
| ENPP2 | C16orf93 |
| ADAMTS5 | C19orf12 |
| SFXN2 | C19orf80 |
| GRHL2 | C1QTNF2 |
| PCCB | C1QTNF7 |
| TSPAN13 | C1R |
| TMEM125 | C1orf198 |
| ARHGEF40 | C1orf50 |
| BCL6 | C1orf64 |
| SLC29A4 | C1orf74 |
| HIST1H4I | C20orf194 |
| FAM13A | C2CD2 |
| GBE1 | C2orf40 |
| FAH | C2orf88 |
| ALDH1A1 | C3 |
| RDH11 | C3orf14 |
| ALDH4A1 | C3orf52 |
| PRG4 | C3orf55 |
| PPP2R2C | C4orf19 |
| AIF1L | C5orf64 |
| HOXC11 | C6orf106 |
| MYO5B | C6orf57 |
| IRAK3 | C6orf99 |
| ZNF687 | C7 |
| GPR153 | C7orf49 |
| DEFB132 | C8orf34 |
| TTC23 | C8orf60 |
| GABRE | C8orf88 |
| SIM1 | CA12 |
| JAG1 | CA3 |
| OLFM1 | CA4 |
| NUAK2 | CABLES2 |
| HIST1H4H | CABYR |
| SHTN1 | CACFD1 |
| MAP7 | CACHD1 |
| NARS2 | CACNA1D |
| BOK | CACNA2D1 |
| CST1 | CACYBP |
| DDR1 | CADM2 |
| RAB11FIP4 | CAHM |
| CRIM1 | CALB2 |
| TMEM273 | CALCA |
| FOXO1 | CALCB |
| BMP8B | CAMK1 |
| ETS2 | CAPN13 |
| WBP11 | CAPN2 |
| ZBTB16 | CAPN6 |
| EVPL | CAPN9 |
| LBH | CAPSL |
| TMEM35B | CARD6 |
| EPB41L4B | CARD8 |
| HOXD9 | CASQ2 |
| HOXB13 | CAT |
| TMEM255A | CAV1 |
| DUSP6 | CAV2 |
| DPYD | CBLN1 |
| PPME1 | CBR4 |
| SLC25A27 | CBX3 |
| SATB1 | CBX4 |
| MYOM1 | CBX7 |
| SLC9A3R1 | CCDC160 |
| NOS3 | CCDC170 |
| SPRY2 | CCDC178 |
| SCARA5 | CCDC3 |
| TTC38 | CCDC50 |
| ACAT1 | CCDC64B |
| CCDC34 | CCDC68 |
| SMPDL3B | CCDC69 |
| CPXM2 | CCDC80 |
| RNPEP | CCDC85A |
| HRCT1 | CCDC92 |
| PPP2R1B | CCL11 |
| ALPK3 | CCL8 |
| CASTOR3 | CD24 |
| ABO | CD300LG |
| TWNK | CD34 |
| CIAO2A | CD36 |
| AVPI1 | CD80 |
| CCNL1 | CD9 |
| ADAMTS18 | CD93 |
| JUP | CDC14B |
| TLE4 | CDC42EP4 |
| NLGN2 | CDC42SE1 |
| DNM1 | CDC45 |
| MRPS23 | CDCA2 |
| PGM5 | CDCA3 |
| PIK3R3 | CDCA4 |
| LRRC59 | CDCA5 |
| ATP6V1G1 | CDCA8 |
| ALDOC | CDCP1 |
| KCNJ16 | CDH1 |
| DSEL | CDH13 |
| NARS | CDH19 |
| FGD5 | CDH3 |
| BICDL1 | CDH5 |
| FA2H | CDIP1 |
| EGR2 | CDK12 |
| MAP1LC3C | CDKN1A |
| DES | CDKN1C |
| SORD | CDKN2B |
| CCL21 | CDKN2C |
| ATP1A2 | CDO1 |
| ACVR1C | CDS1 |
| RTKN | CEACAM6 |
| HAS1 | CEBPA |
| PDK4 | CEBPD |
| RND3 | CELF2 |
| SMKR1 | CEMIP |
| LDB2 | CENPM |
| SIM2 | CENPW |
| LRRK2 | CEP68 |
| EGR1 | CERS2 |
| C6orf132 | CETP |
| FANCF | CFB |
| SEPT4 | CFD |
| BMF | CFH |
| DPT | CFL2 |
| TPPP | CGN |
| RXRA | CH25H |
| GPAT3 | CHD1L |
| FDX1 | CHGB |
| SPAG1 | CHKA |
| SLC7A10 | CHL1 |
| PEA15 | CHRDL1 |
| HEY1 | CHST11 |
| ST6GALNAC6 | CHST7 |
| DLX4 | CIDEA |
| MTCL1 | CIDEC |
| SLC35F6 | CITED2 |
| KDF1 | CKMT2 |
| FIGN | CKS2 |
| EPS8 | CLCN6 |
| HIST1H2AG | CLDN1 |
| TCF7L2 | CLDN11 |
| SMIM3 | CLDN4 |
| BTBD11 | CLDN5 |
| TBX15 | CLDN7 |
| SPRY1 | CLEC10A |
| MLF1 | CLEC3A |
| GDPD5 | CLIC2 |
| OLFML2A | CLIP4 |
| FUT2 | CLMP |
| SOD3 | CLRN3 |
| ANGPTL4 | CLSTN2 |
| SRD5A3 | CLU |
| GOT2 | CNNM4 |
| TRIM62 | CNRIP1 |
| SIPA1L3 | CNTNAP2 |
| EPDR1 | COBL |
| ATAT1 | COBLL1 |
| LGI4 | COG2 |
| GPRC5A | COL11A1 |
| DCLK1 | COL15A1 |
| PSD4 | COL1A1 |
| ITPKB | COL24A1 |
| PJA1 | COL4A1 |
| PTH2R | COL4A2 |
| PAQR5 | COL4A3BP |
| VRK2 | COL6A6 |
| ENO2 | COLCA2 |
| CADM3 | COPG1 |
| C1QTNF7 | COPG2IT1 |
| THSD1 | COPRS |
| PNRC1 | COQ10A |
| PITPNC1 | COQ3 |
| SYNGR3 | CORO2B |
| MARVELD3 | COX14 |
| GDF10 | COX17 |
| HOXA4 | COX6C |
| PPP1R1B | CPED1 |
| MYCT1 | CPM |
| CDS1 | CPNE2 |
| SERINC2 | CPQ |
| MXRA7 | CPS1 |
| HP | CPXM1 |
| PFKFB1 | CRABP2 |
| GIMAP6 | CREB3L4 |
| METTL7A | CREB5 |
| MECOM | CRHBP |
| MPZ | CRIM1 |
| LEP | CRISP3 |
| AKT3 | CRTAP |
| AK3 | CRYAB |
| PDZRN3 | CRYBG3 |
| MSMO1 | CS |
| AASS | CSGALNACT1 |
| HIST3H2A | CSN1S1 |
| NLK | CSPG4 |
| PDHX | CSRNP1 |
| ZBED3 | CSRP2 |
| PKIA | CST6 |
| PLEKHA4 | CST9L |
| GUCY1B1 | CTA-384D8.35 |
| STX11 | CTA-445C9.15 |
| KCTD12 | CTB-167B5.2 |
| ABCC9 | CTD-2325A15.5 |
| TLCD1 | CTNNAL1 |
| HOXA10 | CTNNBIP1 |
| CAT | CTPS2 |
| ADHFE1 | CTSF |
| TK2 | CUTC |
| ABCD2 | CX3CL1 |
| RILP | CXADR |
| CHRDL1 | CXCL1 |
| FAM117A | CXCL12 |
| NYNRIN | CXCL13 |
| BRCC3 | CXCL14 |
| AIFM2 | CXCL2 |
| AVPR2 | CXCL3 |
| GNG2 | CXCR2 |
| APOLD1 | CXCR4 |
| KLHL31 | CYB561 |
| SAA2 | CYB5D2 |
| FRZB | CYP26A1 |
| SNN | CYP26B1 |
| PFKFB4 | CYP27B1 |
| SNX24 | CYP2B7P |
| ZDHHC16 | CYP2J2 |
| MORN3 | CYP46A1 |
| DGAT2 | CYP4B1 |
| MAGEH1 | CYP4Z2P |
| SOX18 | CYS1 |
| DLC1 | CYSTM1 |
| NFATC1 | CYYR1 |
| GPRASP1 | DAAM1 |
| PYGL | DAAM2 |
| NPR2 | DANCR |
| PLIN2 | DAPP1 |
| GIPC2 | DCTPP1 |
| ABCC5 | DCUN1D3 |
| FLI1 | DDIT4L |
| S1PR1 | DDR2 |
| TBKBP1 | DDX43 |
| SH3BP5 | DECR1 |
| HSPB2 | DEDD2 |
| PTPRM | DEFB132 |
| ERBB3 | DENND2A |
| EBF3 | DENND5A |
| LGALS12 | DGAT1 |
| RAP1GAP | DGAT2 |
| PLA2G2A | DHCR24 |
| ARHGEF10 | DHDDS |
| KDM5B | DHFR |
| B3GALNT1 | DHRS2 |
| SNX3 | DHRS3 |
| SGK2 | DHRS9 |
| LPCAT2 | DIAPH2 |
| CBLN1 | DIO2 |
| RASD1 | DIP2C |
| SLCO2A1 | DLC1 |
| ARTN | DLG3 |
| MOCOS | DLG5 |
| TFPI | DLL1 |
| FAM43A | DMRT2 |
| MLLT11 | DNA2 |
| ITIH5 | DNAH5 |
| ALDH1A3 | DNAJC12 |
| ARNT2 | DNAJC22 |
| KL | DNASE1L3 |
| PPP1R15A | DNER |
| C1RL | DNMT3B |
| TNS1 | DOCK11 |
| SELE | DPCD |
| LYVE1 | DPT |
| PRCP | DPYSL2 |
| TLN1 | DSCAM-AS1 |
| SERPINF2 | DUSP22 |
| CIDEA | DUSP6 |
| ATP2C2 | DUSP7 |
| LRP4 | DYNC1LI2 |
| SRP9 | DYNLRB2 |
| F3 | DYNLT1 |
| FADS3 | EBF1 |
| PCDH18 | EBF2 |
| ALDH2 | EBF3 |
| MAFF | ECHDC1 |
| PIP4P2 | ECHDC3 |
| GPAT4 | ECHS1 |
| SP6 | ECM2 |
| PPP1R12B | ECSCR |
| NDNF | EDN3 |
| ADCY3 | EDNRB |
| CCDC43 | EFEMP1 |
| LMO3 | EFEMP2 |
| PDE3B | EFHC1 |
| PKP3 | EFHD1 |
| ODF2 | EFNA1 |
| RAB3IL1 | EFNA4 |
| RGN | EFNA5 |
| TMED3 | EGFL6 |
| GIMAP1 | EGFLAM |
| PLAC9 | EGR1 |
| PRMT5 | EGR2 |
| GRK5 | EGR3 |
| CRNKL1 | EHBP1 |
| GPD1 | EHHADH |
| EFNB1 | EIF1 |
| MFSD4A | EIF2AK1 |
| HSD17B7 | EIF3L |
| RSPO3 | EIF4B |
| ACSS2 | EIF4E3 |
| MATN2 | EIF4EBP2 |
| FBXO17 | EIF5A |
| CYP2U1 | ELAC1 |
| ZNF521 | ELF3 |
| STXBP1 | ELL3 |
| TCN1 | ELOVL2-AS1 |
| PLXNA3 | ELTD1 |
| EXOSC3 | EMCN |
| ADGRF5 | EML1 |
| VAV2 | EMP1 |
| BIN1 | EMP2 |
| DSP | EMX2 |
| PDLIM1 | ENC1 |
| PPP3CC | ENO2 |
| PXDC1 | ENOX1 |
| PDE1B | ENPEP |
| CCL14 | ENPP2 |
| PCDHB8 | ENTPD7 |
| CD163L1 | EPAS1 |
| PDXDC1 | EPB41L2 |
| VEGFB | EPB41L4A-AS2 |
| DMD | EPB41L4B |
| ARID5B | EPB42 |
| CHML | EPCAM |
| KLF4 | EPDR1 |
| ADCY5 | EPHA2 |
| KIAA1522 | EPHA4 |
| ESM1 | EPHX1 |
| C2orf88 | EPN3 |
| CLNS1A | EPPK1 |
| FAT4 | EPRS |
| HSPA9 | EPYC |
| FAM102A | ERBB2 |
| ACSM5 | ERBB3 |
| SYNPO | ERBB4 |
| MAPK8IP2 | ERCC6L |
| ABCA1 | ERG |
| PEG10 | ERH |
| SPX | ERP27 |
| TFB2M | ERV3-2 |
| DAPK2 | ERVMER34-1 |
| CD24 | ESAM |
| NSF | ESYT1 |
| ADAMTSL3 | ETFDH |
| PIK3R1 | ETNK2 |
| PRPF3 | ETS2 |
| ITGB3 | EVA1C |
| PLPP1 | EXO1 |
| SOCS2 | EYA1 |
| PID1 | EZR |
| EPCAM | F11R |
| MAPK13 | F2RL2 |
| ARHGAP31 | F3 |
| STK40 | F8 |
| TSPAN4 | FA2H |
| NR2F2 | FAAH2 |
| RDH5 | FABP4 |
| CYR61 | FABP5 |
| SKAP2 | FADS3 |
| SLC25A18 | FAH |
| LRIG3 | FAM101B |
| ESD | FAM103A1 |
| SMAD9 | FAM107A |
| CD302 | FAM110C |
| SYT7 | FAM117A |
| ALPL | FAM124B |
| PSMD3 | FAM129A |
| FKBP5 | FAM13A |
| VWF | FAM13C |
| RGS5 | FAM149A |
| GOLM1 | FAM150B |
| MAP1B | FAM162B |
| OSBP2 | FAM171A1 |
| PCOLCE2 | FAM174B |
| OAF | FAM189A2 |
| MMRN1 | FAM19A2 |
| LSR | FAM19A5 |
| TBX2 | FAM210B |
| PFKFB3 | FAM213A |
| AMOTL2 | FAM222A |
| RABIF | FAM228B |
| PCK1 | FAM26E |
| HIST1H3H | FAM3B |
| SMYD3 | FAM46C |
| CARD6 | FAM49A |
| BRI3BP | FAM57A |
| MAPKAPK2 | FAM64A |
| CBX6 | FAM83H |
| HABP4 | FAM92A1 |
| INMT | FANCF |
| FAM110D | FANCI |
| SLC16A2 | FANK1 |
| RGCC | FARP1 |
| CIART | FAT2 |
| CRYAB | FBLN2 |
| CLIC5 | FBLN5 |
| PPM1H | FBN2 |
| CYSLTR1 | FBXL7 |
| ICAM2 | FBXO15 |
| FABP5 | FBXO16 |
| MAB21L1 | FBXO17 |
| LRP5 | FCGR1B |
| CDKN2C | FCGR3B |
| MRAP | FCN3 |
| LONRF1 | FEN1 |
| SLC7A11 | FERMT2 |
| DMTN | FES |
| NR3C1 | FFAR4 |
| STRBP | FGD5 |
| TMEM220 | FGF1 |
| MREG | FGF14-AS2 |
| TSPAN2 | FGF2 |
| PTPN1 | FGFBP2 |
| ARHGAP20 | FGR |
| MAN1A1 | FHL1 |
| IQANK1 | FHL5 |
| NLRP1 | FHOD3 |
| CDC42EP2 | FIGN |
| GYG2 | FILIP1 |
| FAM222A | FKBP9 |
| HOXA9 | FLJ33534 |
| SKI | FLJ36848 |
| NCAM1 | FMNL2 |
| MYEOV | FMO2 |
| TMCC3 | FMO3 |
| PECAM1 | FMOD |
| TPSD1 | FN1 |
| ANGPTL1 | FOS |
| ITGA9 | FOSB |
| FAM83G | FOXA1 |
| SLC22A3 | FOXO1 |
| FCER1A | FOXO6 |
| NFIL3 | FREM1 |
| GGCT | FRMD3 |
| TRIB1 | FRMD4A |
| CYFIP2 | FRY |
| LYPD3 | FSTL1 |
| TMEM170B | FTO |
| C12orf49 | FTX |
| CD93 | FUT1 |
| BST1 | FUT4 |
| SELP | FUT8 |
| TESMIN | FXYD3 |
| TUFT1 | FXYD6 |
| GPR157 | FYN |
| ADH1B | FZD2 |
| FOLR2 | FZD4 |
| TGFBR2 | FZD5 |
| GREM2 | G0S2 |
| SLC12A8 | GAB2 |
| PDE3A | GABARAPL1 |
| NUP210 | GALNT15 |
| SEC23B | GALNT6 |
| HAAO | GAS6 |
| ECHDC1 | GATA3 |
| OXER1 | GBE1 |
| SCN4A | GDF10 |
| S100A14 | GDPD5 |
| FZD5 | GFOD1 |
| HMGN1 | GGCT |
| PER1 | GGTA1P |
| PRND | GHR |
| DIO1 | GID4 |
| MT-ND2 | GIMAP1 |
| NMT2 | GIMAP6 |
| TCEAL1 | GIPC2 |
| FCGRT | GJA4 |
| CRTAP | GKAP1 |
| SLC14A1 | GLDN |
| PLAC1 | GLYAT |
| AMZ1 | GLYATL2 |
| RNF180 | GNAI1 |
| PLXNA4 | GNAL |
| DTX1 | GNAZ |
| COPA | GNG11 |
| PGM1 | GNS |
| TCEAL3 | GOLT1A |
| CCDC3 | GOT2 |
| SYN2 | GP2 |
| RAPGEFL1 | GPAM |
| S100P | GPAT2 |
| CDC42EP3 | GPATCH11 |
| SLC16A13 | GPC3 |
| MARVELD2 | GPC6 |
| TNMD | GPD1 |
| ADIPOR1 | GPER1 |
| TTC28 | GPIHBP1 |
| RGS2 | GPM6A |
| TSPAN3 | GPM6B |
| TMEM63B | GPR116 |
| ID1 | GPR133 |
| KRT8 | GPR137C |
| EIF4EBP2 | GPR143 |
| GRB2 | GPR146 |
| SHROOM2 | GPR64 |
| SEMA4A | GPRASP1 |
| CCDC69 | GPRC5A |
| SPARCL1 | GPT2 |
| PIK3C2B | GPX3 |
| SAYSD1 | GREB1 |
| TRO | GRHL2 |
| TSPAN12 | GSN |
| ELF3 | GSTO2 |
| NR3C2 | GTF2H2B |
| HHEX | GUCY1A3 |
| MTMR10 | GULP1 |
| NPR1 | GYG2 |
| EPHA10 | GYS2 |
| SVIP | GZMH |
| PCDHB4 | H2BFS |
| IRS2 | HADH |
| ACLY | HADHB |
| MYADM | HAMP |
| C1orf210 | HAPLN1 |
| ADD3 | HAS1 |
| KANK3 | HBB |
| MGAT3 | HBD |
| IL6 | HCAR3 |
| SYCP2 | HCG11 |
| AFAP1L1 | HDAC4 |
| GPT | HDAC9 |
| MANEAL | HDGF |
| SBK1 | HEBP2 |
| HSP90AB1 | HECW2 |
| MET | HEY1 |
| DAAM2 | HGD |
| ADRB2 | HIPK2 |
| ZNF503 | HIST1H2AE |
| SSPN | HIST1H2BD |
| KCNK1 | HIST1H2BE |
| F10 | HIST1H2BK |
| ANTXR2 | HIST1H3E |
| CCT2 | HIST1H4D |
| RASSF9 | HIST2H2BE |
| LEO1 | HIST3H2A |
| STAT5B | HK1 |
| MORF4L2 | HK2 |
| TRIB3 | HLA-DQB2 |
| MST1R | HLF |
| DBNDD1 | HLX |
| PQLC2L | HMGN2P46 |
| FZD4 | HMGN3 |
| HIST1H2BJ | HN1 |
| RBP4 | HNRNPA1 |
| PPP1R1A | HNRNPH1 |
| GRTP1 | HOGA1 |
| CAMK1 | HOMER1 |
| FBLN5 | HOOK1 |
| ADM5 | HOOK2 |
| GNPNAT1 | HOTAIRM1 |
| HSPB6 | HOXA10 |
| DHRS13 | HOXA3 |
| GPR4 | HOXA5 |
| GEM | HOXA7 |
| PCDHB5 | HOXB7 |
| BMP2 | HOXC10 |
| THNSL1 | HOXC13 |
| TWIST2 | HRASLS5 |
| ABCA8 | HRCT1 |
| ITPRIPL1 | HRH1 |
| TUBB6 | HS3ST3A1 |
| GLDN | HS6ST3 |
| SEC16A | HSBP1 |
| HOXC13 | HSD11B1 |
| RARRES2 | HSD17B7 |
| PRRG4 | HSDL2 |
| PLEK2 | HSPA12A |
| CPQ | HSPA12B |
| PLPP3 | HSPB7 |
| ID3 | HSPB8 |
| BCL9 | HSPH1 |
| AP3M2 | HTRA1 |
| PACSIN1 | HYAL1 |
| PHKA1 | HYOU1 |
| PAK1 | IBSP |
| BSPRY | ICAM2 |
| THBD | ID4 |
| RAMP3 | IER3 |
| TFAP2A | IER5L |
| PLIN1 | IFFO1 |
| MS4A2 | IFI6 |
| IGFBP3 | IGF1 |
| AOX1 | IGSF10 |
| RGL1 | IK |
| FABP4 | IL15RA |
| YEATS4 | IL17D |
| ABI3BP | IL17RB |
| NMNAT2 | IL1R1 |
| SCML1 | IL20 |
| COLEC12 | IL33 |
| SDC1 | IL6 |
| AMOTL1 | IL6R |
| SRP54 | INHBA |
| GSPT1 | INHBB |
| AFAP1L2 | INMT |
| ATIC | INTS8 |
| RASIP1 | IQSEC1 |
| MEOX2 | IRF9 |
| HCAR2 | IRS2 |
| SNRK | IRX3 |
| NCSTN | IRX5 |
| CXCL12 | ISCU |
| HOXD8 | ISG15 |
| AADAC | ITGA1 |
| GPRC5B | ITGA6 |
| EIF2AK1 | ITGA7 |
| NR4A3 | ITGB1BP1 |
| CACNA2D1 | ITGB5 |
| DAP | ITIH3 |
| ATP8A1 | ITIH5 |
| CSN1S1 | ITM2A |
| LLGL2 | ITSN1 |
| ABCA6 | JADE1 |
| GGT5 | JAG2 |
| PMAIP1 | JAM2 |
| MT1X | JPH1 |
| TPSAB1 | JUNB |
| ARAP3 | KANK3 |
| GHR | KANK4 |
| CD34 | KANSL1-AS1 |
| EMP1 | KAT2B |
| MOCS1 | KAT7 |
| SPTBN1 | KATNAL1 |
| AKR1C3 | KBTBD11 |
| FMO3 | KCNA5 |
| NMB | KCNB1 |
| CLDN4 | KCND2 |
| PRRT4 | KCNJ8 |
| PECR | KCNS3 |
| PITX1 | KCTD13 |
| ZNF423 | KDF1 |
| PLTP | KDR |
| COL4A1 | KHDRBS3 |
| CORO2B | KIAA0101 |
| RNASE1 | KIAA0556 |
| LIMS2 | KIAA0907 |
| PLAGL1 | KIAA1024 |
| FAM241B | KIAA1324 |
| XG | KIAA1377 |
| RELN | KIAA1462 |
| CAPN13 | KIF18B |
| ST6GALNAC1 | KIF26A |
| THRSP | KIF26B |
| FGF7 | KL |
| SLC39A11 | KLB |
| HBB | KLF2 |
| IL33 | KLF4 |
| TPPP3 | KLF8 |
| IGFBP6 | KLF9 |
| APOO | KLHDC8B |
| PLCL2 | KLHDC9 |
| PTGFR | KLHL21 |
| CASP6 | KLHL31 |
| NRP1 | KLHL36 |
| TWIST1 | KLRF1 |
| PTX3 | KLRG2 |
| GRB7 | KMO |
| SOBP | KNOP1 |
| ZMYND8 | KRT18 |
| FAM122B | KRT7 |
| DPYSL2 | KRTCAP3 |
| ACE | KYNU |
| GLDC | L3HYPDH |
| POLB | L3MBTL4 |
| KLF6 | LAMA2 |
| MRAS | LAMC1 |
| ESRP2 | LAMP5 |
| PLA2G4A | LARP6 |
| PKD1 | LASP1 |
| PRR15L | LBH |
| ARC | LBR |
| IFNGR1 | LCA5 |
| TMEM241 | LCOR |
| SPTSSA | LCP1 |
| SNTB2 | LDB2 |
| TPMT | LDHB |
| FRAT2 | LDHD |
| DNAJB4 | LEP |
| JDP2 | LEPROT |
| CFD | LGALS12 |
| CSPG4 | LGALS4 |
| ECSCR | LHCGR |
| TSPAN6 | LHFP |
| PRKCH | LHX6 |
| STBD1 | LIFR |
| MUCL1 | LIG1 |
| ADCYAP1R1 | LILRB5 |
| MMRN2 | LIMA1 |
| DKK2 | LIMCH1 |
| RBP7 | LINC00086 |
| INKA2 | LINC00526 |
| TMEM140 | LINC00622 |
| USP14 | LINC00667 |
| HIST1H3G | LINC00702 |
| BTG1 | LINC00888 |
| INPP1 | LINC00893 |
| RIMKLA | LINC00909 |
| ZFP36 | LINC00922 |
| SNCG | LINC00965 |
| VEGFD | LINC00968 |
| LRRN4CL | LINC01140 |
| KCNQ1 | LINC01354 |
| PRKCA | LINC01355 |
| RTN4 | LINC01420 |
| CREB5 | LIPE-AS1 |
| CPM | LIPH |
| CYYR1 | LIX1L |
| SCARA3 | LMCD1 |
| ATF3 | LMO3 |
| JARID2 | LMOD1 |
| SASH1 | LOC100130219 |
| IDH1 | LOC100132735 |
| FBLN7 | LOC100286925 |
| G0S2 | LOC100287387 |
| C1QTNF1 | LOC100288860 |
| HRASLS5 | LOC100288911 |
| KHDC4 | LOC100505715 |
| PLS1 | LOC100506022 |
| SPATA17 | LOC100506098 |
| ERRFI1 | LOC100506325 |
| RNLS | LOC100506388 |
| CDH5 | LOC101060264 |
| KAT2B | LOC101060391 |
| ANKRD29 | LOC101926960 |
| LRRC20 | LOC101927263 |
| ZDHHC2 | LOC101927752 |
| LRIF1 | LOC101927841 |
| MAP3K8 | LOC101927943 |
| ACSL1 | LOC101928076 |
| MYZAP | LOC101928173 |
| TMTC1 | LOC101928245 |
| MGST1 | LOC101928370 |
| SGCG | LOC101929504 |
| RERGL | LOC101929787 |
| CLEC14A | LOC101930114 |
| SH2B3 | LOC102723493 |
| PLXNA2 | LOC102723845 |
| TTLL12 | LOC102723927 |
| PENK | LOC102724356 |
| C1QTNF2 | LOC153546 |
| STAT5A | LOC153577 |
| KLHL21 | LOC154761 |
| ROBO4 | LOC284578 |
| RIPOR1 | LOC284825 |
| ABCA5 | LOC285812 |
| CCDC88C | LOC286367 |
| INKA1 | LOC401052 |
| CALHM2 | LOC439938 |
| TDRKH | LOC644656 |
| MSRA | LOC728392 |
| RAB25 | LOC728819 |
| FGFBP2 | LOC729970 |
| ABHD6 | LOH12CR2 |
| CDH1 | LPAR1 |
| MAP3K20 | LPHN3 |
| TMEM14A | LPIN1 |
| LMO2 | LPL |
| IQCG | LPP-AS2 |
| CCDC134 | LRFN5 |
| BMP6 | LRG1 |
| F8 | LRP1B |
| LPL | LRRC15 |
| CTBP2 | LRRC32 |
| RRAS2 | LRRC34 |
| KLHL38 | LRRC3B |
| FKBP4 | LRRC8E |
| IRX5 | LRRN3 |
| RAD51C | LRRN4CL |
| PLEKHG5 | LSS |
| PARD6B | LY86 |
| MAMLD1 | LYPD3 |
| VSIR | LYPD6B |
| CNTFR | LYRM1 |
| TF | LYRM7 |
| PIGO | LYSMD4 |
| GPER1 | LYVE1 |
| GRK3 | MAGED2 |
| CYP27B1 | MAGEL2 |
| GABARAPL1 | MAGI2 |
| PTPRS | MAGI2-AS3 |
| GSPT2 | MAL |
| NR4A2 | MALL |
| IQSEC1 | MAMDC2 |
| EPRS | MAML2 |
| STX3 | MAMLD1 |
| RHOQ | MAN1C1 |
| BROX | MAN2A2 |
| PPP1R16B | MANEAL |
| ZC3H12C | MANF |
| ETFDH | MAOA |
| FAM89A | MAOB |
| NAP1L2 | MAP1LC3C |
| CPA3 | MAP3K1 |
| TMEM132C | MAP3K5 |
| ALDH1L1 | MAP3K9 |
| PTGS2 | MAPK10 |
| SGCE | MARC1 |
| LEPROT | MARC2 |
| ITGB1BP1 | MARCH3 |
| SVEP1 | MARCKSL1 |
| SELENOI | MARCO |
| CRABP2 | MARVELD2 |
| MFAP4 | MARVELD3 |
| IBSP | MAST3 |
| HK1 | MAT2A |
| CD209 | MB |
| AOC3 | MBNL1-AS1 |
| GPR143 | MBOAT2 |
| PDGFD | MBOAT7 |
| ZFP36L1 | MCAM |
| CLIC2 | MCCC1 |
| WWC1 | MDFIC |
| BCL2L2 | ME1 |
| KCNJ2 | MED1 |
| PAX9 | MEF2C |
| TLCD2 | MEGF10 |
| SLC12A7 | MEOX1 |
| HSDL2 | MEOX2 |
| TBC1D30 | MEST |
| MAZ | MFAP4 |
| SIRPA | MGC45800 |
| TRPS1 | MGLL |
| CEBPD | MGP |
| NOTCH4 | MGST1 |
| NNAT | MICAL2 |
| HIST3H2BB | MICU3 |
| PDZD2 | MID1 |
| TIMP4 | MID1IP1 |
| C2orf15 | MID2 |
| USP43 | MIR143HG |
| IARS2 | MKI67 |
| SEC11C | MLEC |
| HPGDS | MLIP |
| SULT1C4 | MLLT11 |
| GSTM5 | MLPH |
| ACADS | MMD |
| KLF13 | MME |
| EIF4A3 | MMP1 |
| HPD | MMP11 |
| TTYH2 | MMP28 |
| RBPMS2 | MMRN1 |
| CD300LG | MMRN2 |
| HSPA8 | MOAP1 |
| CFI | MOCS1 |
| FGFRL1 | MPDZ |
| MRPS35 | MPHOSPH6 |
| TNFRSF10D | MPP1 |
| SLIT2 | MPP6 |
| NRIP2 | MRAP |
| JPT2 | MRAP2 |
| HIST1H2AI | MRAS |
| SOX12 | MRC1 |
| PPP1R14A | MRGBP |
| CD200 | MRGPRF |
| CES1 | MRPL48 |
| SORBS1 | MRPL51 |
| SYNE1 | MRPS15 |
| MED24 | MRPS23 |
| NACC2 | MRVI1 |
| SLC23A1 | MSL1 |
| MAOA | MSMB |
| ERMP1 | MSRB2 |
| MAB21L4 | MSRB3 |
| PYGM | MSX1 |
| LRP11 | MT1M |
| CLEC3B | MTCL1 |
| EPB41L2 | MTFP1 |
| KLHL36 | MTHFD1 |
| UGP2 | MTMR10 |
| CDKN1C | MTURN |
| DDR2 | MTUS1 |
| PLA2R1 | MUC1 |
| SH3D19 | MUM1 |
| ARHGEF17 | MXI1 |
| LRRFIP2 | MXRA5 |
| NT5C3A | MXRA7 |
| GADD45A | MYADM |
| BAALC | MYB |
| HIST1H2BK | MYCT1 |
| PPARG | MYEOV |
| TACR1 | MYH1 |
| PROCR | MYH10 |
| RAB15 | MYH11 |
| GYPC | MYLIP |
| PRUNE1 | MYO16 |
| LTBP4 | MYO6 |
| ARHGAP23 | MYOCD |
| VIM | MYOM1 |
| SERP1 | MYOM2 |
| KLHL12 | MYRIP |
| GNAI1 | MYZAP |
| KCNB1 | NAA25 |
| CHMP4C | NAGPA |
| FMO2 | NANOS1 |
| VDAC3 | NAP1L5 |
| BHLHE41 | NAT10 |
| SAMD4A | NAT8L |
| RAB6B | NCALD |
| F2RL3 | NCAPH |
| TMEM41B | NDN |
| SAV1 | NDNF |
| SEMA6A | NDRG2 |
| CABYR | NDRG4 |
| NLGN4X | NDUFS2 |
| NTRK3 | NEFH |
| COL6A6 | NEGR1 |
| UST | NEK10 |
| SLCO4A1 | NELL2 |
| GPAM | NEURL1B |
| CYGB | NEXN |
| ANXA5 | NFIA |
| GSN | NFIB |
| ABCA10 | NFKBIZ |
| NFKBIZ | NIPSNAP3B |
| ZFP36L2 | NKX3-2 |
| SCN1B | NLGN1 |
| ACKR3 | NLGN4X |
| FOSB | NMNAT2 |
| SDC3 | NMT2 |
| HOXC10 | NMU |
| ERO1A | NOSTRIN |
| PHLDB1 | NOV |
| OTUD1 | NPM1 |
| ILDR1 | NPR1 |
| ETS1 | NPR2 |
| OGN | NPR3 |
| LIFR | NPY1R |
| PTPN21 | NPY5R |
| CMA1 | NR1H3 |
| RAB3IP | NR2C2AP |
| KRTCAP3 | NR2F1-AS1 |
| CRTAC1 | NR3C1 |
| LHFPL6 | NR3C2 |
| IRX6 | NR4A3 |
| C11orf95 | NRIP2 |
| TMA16 | NRIP3 |
| ANKEF1 | NRN1 |
| MAST2 | NRP1 |
| PRUNE2 | NRROS |
| PEMT | NTRK2 |
| TYRO3 | NUAK1 |
| MEDAG | NUDT6 |
| CHL1 | NUDT7 |
| P4HA1 | NUP210 |
| LEF1 | NUSAP1 |
| LRRN3 | NWD2 |
| TLE1 | NYNRIN |
| IRF6 | OASL |
| CA3 | OCIAD2 |
| JUNB | OLFM1 |
| STEAP4 | OLFML1 |
| IGSF3 | OLFML2A |
| TOMM34 | OPLAH |
| PLIN4 | OPTN |
| CD36 | OR51E1 |
| NUP62CL | ORC6 |
| GTF2IRD1 | OSBPL1A |
| VPS37C | OSR1 |
| ERVMER34-1 | OVOL1 |
| CNR1 | OVOL2 |
| ACAA2 | OXCT1 |
| FRMD3 | OXLD1 |
| MARK1 | P2RY12 |
| PI16 | P2RY14 |
| CTSG | PAFAH1B3 |
| TRAP1 | PAK3 |
| SUN2 | PAK6 |
| LAMA3 | PALM |
| SLC37A1 | PALM2 |
| RNF125 | PALMD |
| FHL5 | PAM |
| NECTIN4 | PAN3-AS1 |
| LIMCH1 | PARM1 |
| RETSAT | PARVA |
| P2RY12 | PAX8-AS1 |
| CDIP1 | PC |
| CEACAM6 | PCAT19 |
| DRAM2 | PCDH18 |
| ZMPSTE24 | PCDH19 |
| SAA2-SAA4 | PCDH9 |
| VEGFA | PCDHB15 |
| COG2 | PCDHB7 |
| CD59 | PCK1 |
| LIPE | PCOLCE2 |
| FAM69A | PCSK5 |
| TUBB2A | PCYOX1 |
| SIK1B | PDE2A |
| ZFAND5 | PDE3B |
| KLF9 | PDE8B |
| CIDEC | PDGFD |
| CACNB3 | PDGFRB |
| SIAH2 | PDHA1 |
| SLC1A4 | PDIA4 |
| MAP7D3 | PDK3 |
| MTURN | PDP2 |
| ADAM33 | PDRG1 |
| NEURL1B | PDZD2 |
| CDO1 | PDZRN3 |
| NIPSNAP1 | PDZRN3-AS1 |
| GIMAP8 | PDZRN4 |
| C2orf74 | PEAR1 |
| SFXN1 | PECAM1 |
| VLDLR | PEG10 |
| AAGAB | PEG3-AS1 |
| GATA6 | PELI2 |
| PHLDB2 | PEMT |
| FANCL | PET112 |
| LIX1L | PEX11A |
| C6 | PEX19 |
| FNDC5 | PFKFB3 |
| PRELP | PGAP2 |
| HDAC1 | PGM1 |
| EPHA4 | PGM5-AS1 |
| CEP70 | PHGDH |
| CRMP1 | PHYHD1 |
| RBMS3 | PIAS3 |
| CH25H | PIGZ |
| NR4A1 | PIP |
| CNRIP1 | PITPNA-AS1 |
| NPY1R | PJA1 |
| EPN3 | PKDCC |
| TPSB2 | PKIG |
| FSTL4 | PKM |
| SORBS3 | PKN3 |
| PAPLN | PLA2G4A |
| GRASP | PLAC9 |
| ANXA1 | PLAU |
| VDAC1 | PLAUR |
| TLL1 | PLCL2 |
| AVPR1A | PLEK2 |
| RHOU | PLEKHA6 |
| C15orf48 | PLEKHA7 |
| FAM49A | PLEKHA8P1 |
| NPY5R | PLEKHH2 |
| MRPL49 | PLEKHM3 |
| ADM2 | PLIN1 |
| DNASE1L3 | PLIN4 |
| ACVRL1 | PLN |
| GGPS1 | PLP1 |
| CACHD1 | PLSCR4 |
| NEURL1 | PLXNA4 |
| HSPA12B | PMEPA1 |
| SDHD | PMP2 |
| ANO6 | PNMAL2 |
| PRR36 | POC1A |
| TPBG | PODXL |
| XPNPEP2 | POLQ |
| ANXA6 | POLR1C |
| APOD | POLR3GL |
| SYNE3 | POLR3K |
| SLC25A20 | POPDC3 |
| PEAR1 | POU2F3 |
| HAPLN1 | PPAP2A |
| HRC | PPAP2B |
| GULP1 | PPAPDC1A |
| TNNT3 | PPAPDC1B |
| TMEM88 | PPAPDC3 |
| LDHA | PPARG |
| RASL12 | PPEF1 |
| MLXIPL | PPFIA1 |
| SLC20A1 | PPIH |
| KBTBD11 | PPL |
| ABHD17C | PPM1L |
| C7 | PPP1CA |
| MYCBP | PPP1R14A |
| CYB5R3 | PPP1R14C |
| FXYD6 | PPP1R15A |
| CPS1 | PPP1R16B |
| NAT8L | PPP1R1A |
| KCNAB1 | PPP1R36 |
| CNDP2 | PPP2R1B |
| AGPAT2 | PRDX6 |
| TMOD1 | PRELP |
| FAM166B | PRG4 |
| TMEM199 | PRICKLE2 |
| ZNF75A | PRKAR2B |
| WNT11 | PRKCZ |
| DENND2A | PRKD1 |
| RASL10B | PROCR |
| HSH2D | PROK2 |
| C20orf194 | PROM2 |
| BOC | PROS1 |
| PTH1R | PRPH2 |
| ALDH1A2 | PRR15 |
| MRC1 | PRR15L |
| PIP5K1B | PRRG3 |
| RPS6KA2 | PSMD9 |
| CRB3 | PSRC1 |
| TMEM200B | PTEN |
| NDRG2 | PTGER3 |
| RRAGD | PTGS2 |
| PCDHB14 | PTH1R |
| MAN2A2 | PTH2R |
| KCNJ8 | PTK6 |
| RRAD | PTN |
| B4GALT1 | PTPDC1 |
| ITM2A | PTPLA |
| PALMD | PTPN14 |
| TMEM37 | PTPN6 |
| DAB2IP | PTPRB |
| FAAH2 | PTPRM |
| REM1 | PTPRN2 |
| NIPSNAP3B | PTPRZ1 |
| BAIAP2L1 | PTRF |
| MARCKSL1 | PTRH2 |
| ADCY4 | PTTG1 |
| NUCB2 | PTTG3P |
| SOCS3 | PUS7L |
| PKN3 | PVRL3 |
| HIST1H2AC | PVRL4 |
| OSR2 | PXDN |
| TSPAN7 | PXK |
| VDR | PXMP2 |
| ARID5A | PYCR1 |
| UTP18 | PYGL |
| TEF | PYGM |
| LYSMD1 | QDPR |
| GALNT17 | QPRT |
| COX4I2 | R3HDM4 |
| PPT1 | RAB11FIP1 |
| ZBTB47 | RAB11FIP4 |
| NR2F1 | RAB15 |
| AKR1C1 | RAB17 |
| C2CD2 | RAB7B |
| PTGER4 | RABIF |
| ERG | RAD54L |
| RCBTB2 | RADIL |
| HIST1H2BC | RAI2 |
| THRA | RAP2B |
| DLG3 | RASD1 |
| COL14A1 | RASEF |
| NAA20 | RASGEF1A |
| SDK1 | RASGRF2 |
| RCAN2 | RASGRP1 |
| GALNT12 | RASIP1 |
| ELOVL3 | RASL12 |
| FBXO27 | RASSF9 |
| DLL4 | RBM39 |
| ESRP1 | RBMS1 |
| LEFTY2 | RBMS3 |
| MEST | RBP4 |
| EPAS1 | RBP7 |
| HAUS1 | RBPMS |
| ERBB2 | RBPMS-AS1 |
| DNAJC22 | RBPMS2 |
| FGF2 | RCAN2 |
| RNF150 | RDH5 |
| CCDC50 | REEP5 |
| HEPACAM | RERGL |
| PROM2 | RET |
| BIK | RETSAT |
| SLC19A3 | RGCC |
| BMP5 | RGN |
| EVA1C | RGS17 |
| SLC2A4 | RGS22 |
| BHMT2 | RGS3 |
| DOK5 | RGS4 |
| LGALS3 | RGS7BP |
| HIST1H2BD | RHOBTB3 |
| BPNT1 | RHOH |
| ZNF502 | RHOQ |
| MSX1 | RHPN1-AS1 |
| GJA4 | RHPN2 |
| AQP7 | RILPL2 |
| FHL1 | RIPK4 |
| ADIPOQ | RMDN3 |
| PTK6 | RMI2 |
| P2RY14 | RNASE4 |
| CD99L2 | RNF150 |
| KIF1C | RNF180 |
| ZBTB2 | RNF182 |
| MCAM | RNF2 |
| PRIMA1 | RNF213 |
| SCUBE3 | RNF24 |
| ZNF662 | RNLS |
| PCCA | ROPN1 |
| LOXL4 | ROPN1B |
| MT-ND1 | RORB |
| LZTS1 | RP1-193H18.2 |
| TMEM204 | RP11-116O18.1 |
| PLK3 | RP11-124L9.5 |
| PROX1 | RP11-1275H24.2 |
| TINCR | RP11-174G6.5 |
| TMEM132A | RP11-180N14.1 |
| TMEM62 | RP11-199F11.2 |
| CFLAR | RP11-21L23.2 |
| LDHB | RP11-245J9.5 |
| SNCAIP | RP11-247L20.4 |
| JUN | RP11-248J18.2 |
| UBE2E2 | RP11-295M18.6 |
| TMEM209 | RP11-2E11.9 |
| SPNS2 | RP11-305K5.1 |
| GNG11 | RP11-305O6.3 |
| CTTNBP2 | RP11-330O11.3 |
| HSD11B1 | RP11-355B11.2 |
| PTBP3 | RP11-378A13.1 |
| SRSF8 | RP11-37C7.3 |
| CYTL1 | RP11-389C8.2 |
| MB | RP11-401P9.4 |
| PRKAR2B | RP11-44F14.8 |
| DPP3 | RP11-506O24.2 |
| SYT11 | RP11-532F12.5 |
| SPAAR | RP11-585P4.5 |
| ACACB | RP11-589P10.5 |
| COPB2 | RP11-5C23.1 |
| PRELID3B | RP11-736K20.5 |
| PKDCC | RP11-747H7.3 |
| PCDHB16 | RP11-846E15.2 |
| KRT80 | RP11-932O9.10 |
| GPC3 | RP11-96D1.11 |
| MPPED2 | RP13-270P17.3 |
| ABCG2 | RP3-406A7.7 |
| SLIT3 | RP4-612B15.3 |
| ATP6AP1 | RP4-635E18.8 |
| CPNE2 | RPL22L1 |
| PNPLA2 | RPL26L1 |
| ARRDC4 | RPS21 |
| FGF18 | RPS24 |
| SYNPO2 | RPS3 |
| GPX3 | RPS6KA1 |
| TIFA | RRAD |
| WNT7B | RRAGD |
| IGFBP7 | RRAS2 |
| MEGF6 | RRS1 |
| ACSS3 | RSPH1 |
| SEMA6C | RSPO3 |
| CCND2 | RTN3 |
| KANK1 | RUNDC3B |
| NKAIN1 | RUNX1T1 |
| RASA3 | RWDD1 |
| ABCC6 | S100A10 |
| FNDC4 | S100A14 |
| PPM1E | S100B |
| MAGEF1 | S100G |
| ARHGEF15 | S100P |
| ABLIM1 | S1PR1 |
| FOXO4 | S1PR3 |
| TCEAL4 | SAMD12 |
| PELI2 | SAMD4A |
| CERS2 | SAMD9 |
| MMD | SAPCD2 |
| CXCL2 | SART1 |
| POGK | SASH1 |
| CD9 | SBK1 |
| ADAMTS10 | SCARA5 |
| CALB2 | SCD |
| JADE1 | SCGB1D2 |
| SLC16A7 | SCGB2A2 |
| FXYD1 | SCMH1 |
| OVOL2 | SCN3A |
| PYCR1 | SCN4A |
| PXN | SCN4B |
| SLC29A1 | SDC1 |
| SHISA9 | SDK1 |
| ASPA | SDPR |
| LARP6 | SDS |
| FBN2 | SEC14L1 |
| ANKRD65 | SEC61A2 |
| HBEGF | SEC62 |
| PLEKHG6 | SECISBP2 |
| RBBP7 | SECISBP2L |
| ANK2 | SEL1L2 |
| WASF3 | SELE |
| ESYT1 | SELENBP1 |
| NATD1 | SELP |
| TLE2 | SEMA3G |
| PDE8B | SEMA4B |
| LRRC1 | SEMA4D |
| UBE2O | SEMA6A |
| TEK | SEPT11 |
| ANGPT1 | SEPT3 |
| PLP1 | SERPINA1 |
| PRXL2A | SERPINA3 |
| STARD8 | SERPINF1 |
| SH3BGRL2 | SERPING1 |
| PCSK5 | SESTD1 |
| CA4 | SETD7 |
| CAPN5 | SFMBT2 |
| ITSN1 | SFN |
| EIF2S3 | SFRP1 |
| TBC1D2B | SFT2D2 |
| MDFIC | SFXN1 |
| LIG1 | SFXN2 |
| PLPP2 | SGCB |
| CLDN7 | SGCD |
| ACO1 | SGCG |
| ARHGEF28 | SGK2 |
| CLIP3 | SH3BGRL2 |
| FAM222B | SH3BP4 |
| SCARF1 | SH3BP5 |
| ARF3 | SH3KBP1 |
| EEPD1 | SH3RF3 |
| ADAMTSL4 | SHANK2 |
| TDRD5 | SHANK3 |
| CLIP4 | SHE |
| EPHX1 | SHISA9 |
| DEPP1 | SHMT2 |
| FREM1 | SHROOM3 |
| ANPEP | SIK2 |
| CDCP1 | SIM1 |
| HOXA3 | SIX4 |
| TINAGL1 | SKA2 |
| VIT | SKA3 |
| ANXA3 | SKI |
| IL11RA | SLC12A2 |
| HIST1H2AD | SLC12A8 |
| SPART | SLC14A1 |
| TSPAN11 | SLC15A2 |
| SOX17 | SLC16A2 |
| HCN3 | SLC16A7 |
| CRYBG3 | SLC19A3 |
| AKR1C2 | SLC1A4 |
| MBOAT2 | SLC20A1 |
| SYAP1 | SLC24A2 |
| GTF3C1 | SLC24A3 |
| RAB7B | SLC25A14 |
| SYDE1 | SLC25A18 |
| FAXDC2 | SLC25A33 |
| NPR3 | SLC25A51 |
| GLYAT | SLC27A1 |
| CYP4F12 | SLC29A3 |
| CXorf36 | SLC2A1 |
| MYOC | SLC35G2 |
| LRRC42 | SLC38A1 |
| NEDD9 | SLC39A4 |
| HHIPL2 | SLC3A2 |
| GPATCH2 | SLC41A1 |
| HADH | SLC4A4 |
| FBXO16 | SLC4A7 |
| MYOM2 | SLC4A8 |
| HIC1 | SLC50A1 |
| ADGRL4 | SLC7A10 |
| C19orf12 | SLC7A11 |
| CDKN2B | SLC7A6OS |
| CCM2L | SLC9A3R1 |
| FOXN3 | SLCO3A1 |
| SEC14L1 | SLIT3 |
| S100B | SLITRK6 |
| SOX7 | SLX4IP |
| HSPA12A | SMA4 |
| HYAL1 | SMAD9 |
| GIMAP7 | SMARCC1 |
| ZNF146 | SMEK1 |
| KCNIP2 | SMG8 |
| NVL | SMIM3 |
| DOCK11 | SMYD3 |
| FXYD3 | SMYD4 |
| PSKH1 | SNHG18 |
| LHX6 | SNORA21 |
| PREX2 | SNORD89 |
| CSGALNACT1 | SNRPD3 |
| ITPRIP | SNRPE |
| BDKRB2 | SNRPF |
| PPM1F | SNX27 |
| PLPP5 | SOCS2 |
| NUDT7 | SORBS1 |
| MT1A | SORD |
| H3F3A | SORL1 |
| MAF | SORT1 |
| TOP1 | SOX17 |
| TTC9 | SOX18 |
| NFIX | SOX4 |
| CKMT1A | SOX7 |
| ATP2B4 | SP3 |
| PRSS8 | SPAG4 |
| GALNT15 | SPARCL1 |
| SEMA4B | SPATA22 |
| AKAP12 | SPCS3 |
| GOLT1A | SPHK1 |
| SLC35G2 | SPINT1 |
| BUB3 | SPINT2 |
| HBA2 | SPRY1 |
| PLVAP | SPRY2 |
| PLEKHH2 | SPSB1 |
| ARF4 | SPTBN1 |
| PC | SPX |
| KDR | SRP54 |
| CREB3L4 | SRPK1 |
| SHANK3 | SRPX |
| CTNNAL1 | SRSF1 |
| OSBPL1A | SRSF6 |
| ADAMTS1 | SS18L2 |
| HSPA4 | SSPN |
| STOX1 | SSR2 |
| TLR3 | ST14 |
| MT-ND6 | ST3GAL5 |
| CSRNP1 | ST6GALNAC2 |
| KLF8 | ST6GALNAC3 |
| MEOX1 | STAP2 |
| ADRA1B | STARD9 |
| PDLIM3 | STAT5A |
| DOC2B | STC2 |
| PHKG1 | STEAP1 |
| KCNS3 | STEAP4 |
| ADM | STIP1 |
| HOXA7 | STOM |
| HIBADH | STON1 |
| GNAL | STOX1 |
| NOV | STOX2 |
| EGFLAM | STRA13 |
| TRIM68 | STRADB |
| FHDC1 | STS |
| GPIHBP1 | STX11 |
| FLT4 | STX12 |
| PIP5K1A | STXBP1 |
| MAP6D1 | STYK1 |
| CASQ2 | STYXL1 |
| MGLL | SUCNR1 |
| MARK2 | SUSD4 |
| ADGRL2 | SVIP |
| TXNIP | SYAP1 |
| GFOD1 | SYN2 |
| CKMT2 | SYNM |
| IER3IP1 | SYNPO2 |
| CGN | SYT13 |
| NOL4L | SYTL5 |
| TNXB | TADA2A |
| EARS2 | TBC1D4 |
| MME | TBX15 |
| STON1 | TCEAL3 |
| DHCR24 | TCF4 |
| CKMT1B | TCF7L1 |
| GAB2 | TCF7L2 |
| ZNF677 | TCN2 |
| SDC2 | TCTEX1D2 |
| CLDN5 | TDG |
| ENG | TEK |
| WDR45B | TENC1 |
| HOOK1 | TENM4 |
| STT3A | TES |
| PCDH12 | TF |
| MAL2 | TFAP2A |
| DOP1B | TFAP2C |
| ATOH8 | TFCP2L1 |
| HIST1H3D | TFPI |
| NOTCH1 | TGFBR1 |
| TGIF1 | TGFBR2 |
| FMO4 | TGFBR3 |
| RASL11A | THBD |
| TMCO1 | THBS2 |
| SLC6A9 | THBS4 |
| TFPI2 | THRB |
| FOS | THRSP |
| HSD17B11 | THSD4 |
| PTGIS | THSD7A |
| EDN1 | THSD7B |
| SUSD4 | TIE1 |
| EPB41L3 | TIGD1 |
| ALS2CL | TIMP4 |
| HIGD1B | TIPRL |
| SYT13 | TJP1 |
| CAV2 | TK1 |
| SIX4 | TK2 |
| CYP26B1 | TLCD2 |
| TMBIM1 | TLE1 |
| FERMT2 | TLE2 |
| KLF10 | TLN2 |
| TRARG1 | TLR4 |
| BMX | TM2D2 |
| LRRC8C | TM4SF18 |
| OVOL1 | TM7SF2 |
| HOXA5 | TMC4 |
| BCL6B | TMCC3 |
| ABHD15 | TMEM100 |
| MEIS2 | TMEM120B |
| SLC9A9 | TMEM125 |
| EGR3 | TMEM129 |
| KIF26A | TMEM132C |
| LIG3 | TMEM133 |
| PTPRB | TMEM140 |
| P4HA2 | TMEM154 |
| CLSTN1 | TMEM161B-AS1 |
| ZNF676 | TMEM170B |
| C2CD4B | TMEM182 |
| AK4 | TMEM200B |
| RIMKLB | TMEM220 |
| CPNE8 | TMEM246 |
| LILRB5 | TMEM261 |
| SHMT1 | TMEM35 |
| BTNL9 | TMEM37 |
| PIGZ | TMEM38A |
| TRAF7 | TMEM39B |
| PCDHGB7 | TMEM45B |
| FAM107A | TMEM47 |
| OCIAD2 | TMEM51 |
| TMEM254 | TMEM55A |
| TNIP1 | TMEM56 |
| COBLL1 | TMEM79 |
| FOXK2 | TMEM97 |
| EHHADH | TMOD1 |
| TM4SF18 | TMPRSS3 |
| KIAA0895 | TMSB15B |
| ANXA11 | TMSB4X |
| CLDN15 | TMTC1 |
| CORO2A | TNFRSF12A |
| AP1M2 | TNFSF11 |
| RAP1GDS1 | TNFSF13 |
| ANKRD35 | TNIK |
| TGFBR3 | TNMD |
| NOMO1 | TNN |
| CTIF | TNS1 |
| NES | TOB2 |
| CDR2 | TOMM34 |
| FRMD4A | TOP1 |
| SLC52A3 | TOX2 |
| MIPEP | TP73-AS1 |
| SLC25A25 | TPST1 |
| ATP1B2 | TPST2 |
| ADH1C | TPX2 |
| EMCN | TRAF4 |
| FGFR1 | TRAM2-AS1 |
| PARM1 | TRDN |
| BCHE | TRHDE |
| IL3RA | TRHDE-AS1 |
| ZNF165 | TRIM11 |
| LIF | TRIM37 |
| MYRIP | TRIM68 |
| ELMOD3 | TRIT1 |
| PLSCR4 | TRPS1 |
| FLNC | TSHZ2 |
| RHOJ | TSLP |
| HIST2H2BE | TSPAN1 |
| PROK1 | TSPAN11 |
| TACC1 | TSPAN12 |
| HIST1H2AE | TSPAN13 |
| ADAMTS9 | TSPAN18 |
| BEND7 | TSPAN3 |
| MEF2C | TSPAN4 |
| ECHDC3 | TSPAN7 |
| C14orf180 | TSPAN8 |
| RBMS2 | TSR2 |
| SRPX | TST |
| CFL2 | TSTD1 |
| H6PD | TTC27 |
| ACADL | TTC28 |
| PDE2A | TTC39A |
| KRT18 | TTC7B |
| ALDH18A1 | TTC9 |
| ABCA9 | TTLL12 |
| ARHGEF6 | TUBB2A |
|  | TUBB2B |
|  | TUBB6 |
|  | TUBG2 |
|  | TUSC5 |
|  | TWIST1 |
|  | TWIST2 |
|  | TXNIP |
|  | TYRO3 |
|  | UBA2 |
|  | UBAP1 |
|  | UBAP2L |
|  | UBE2C |
|  | UBE2L6 |
|  | UBE2R2 |
|  | UBE2Z |
|  | UGP2 |
|  | UHRF1 |
|  | UNC5CL |
|  | USP13 |
|  | USP33 |
|  | USP46-AS1 |
|  | UVRAG |
|  | VDR |
|  | VEGFB |
|  | VGLL3 |
|  | VKORC1L1 |
|  | VLDLR |
|  | VPS51 |
|  | VSIG4 |
|  | VTI1B |
|  | VWF |
|  | WASF3 |
|  | WBP1L |
|  | WBP4 |
|  | WDFY3-AS2 |
|  | WDR52 |
|  | WDR53 |
|  | WDR5B |
|  | WEE1 |
|  | WFDC1 |
|  | WFS1 |
|  | WHAMM |
|  | WIF1 |
|  | WISP2 |
|  | WNT2 |
|  | WT1 |
|  | WTIP |
|  | XBP1 |
|  | XKR4 |
|  | XPO1 |
|  | XRCC6BP1 |
|  | YBX3 |
|  | YIPF1 |
|  | YPEL4 |
|  | YTHDF1 |
|  | YWHAG |
|  | ZAK |
|  | ZBED3 |
|  | ZBTB16 |
|  | ZBTB20 |
|  | ZCCHC24 |
|  | ZDHHC13 |
|  | ZDHHC16 |
|  | ZEB2 |
|  | ZFAS1 |
|  | ZFHX4-AS1 |
|  | ZFP2 |
|  | ZFP30 |
|  | ZFP36 |
|  | ZG16B |
|  | ZIC2 |
|  | ZMAT2 |
|  | ZMYND12 |
|  | ZNF106 |
|  | ZNF137P |
|  | ZNF204P |
|  | ZNF25 |
|  | ZNF300P1 |
|  | ZNF34 |
|  | ZNF423 |
|  | ZNF518B |
|  | ZNF587 |
|  | ZNF606 |
|  | ZNF667-AS1 |
|  | ZNF75A |
|  | ZSCAN16 |
|  | ZYG11A |
