# Supplementary material for: The Novel Methylation Biomarker NPY5R Sensitizes Breast Cancer Cells to Chemotherapy
Source: Front Cell Dev Biol. 2022 Jan 11;9:798221. doi: 10.3389/fcell.2021.798221 (PMC8787223; doi:10.3389/fcell.2021.798221)
Supplement: Supplementary file 3 [file DataSheet1.pdf]

Supplementary Figures and Figure Legends:

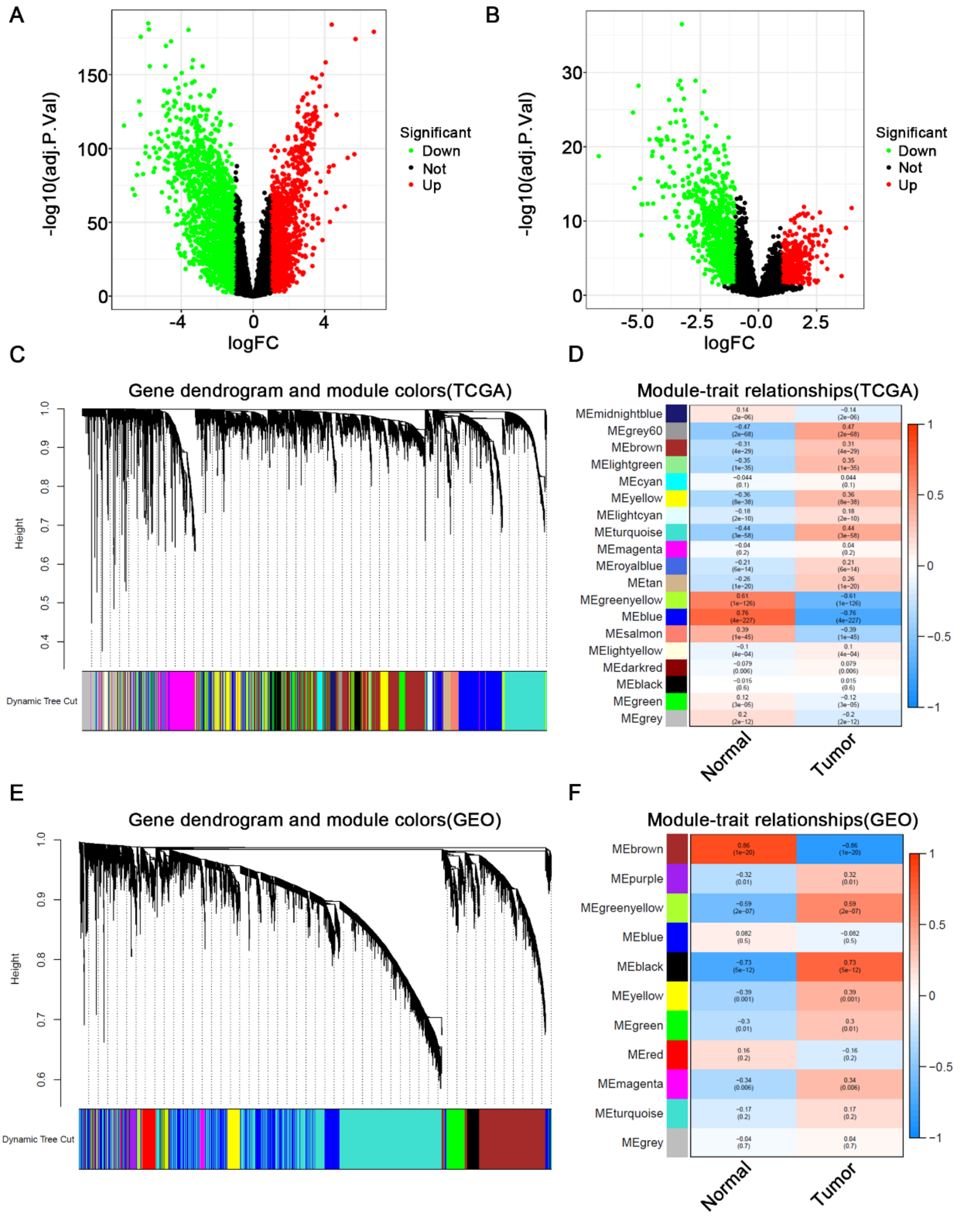

Supplementary Figure 1. Identification of key tumor-related modules via the WGCNA. (a, b) Volcano plot of DEGs in datasets TCGA-BRCA and GSE29431. Red dots represent the genes that were significantly upregulated in tumor samples. Green dots represent the genes that were significantly downregulated in tumor samples. The dotted vertical lines indicate the significance thresholds filter. (c, e) The cluster dendrogram of genes in the key tumor-related modules. (d, f) The module–trait relationship of key tumor-related modules.

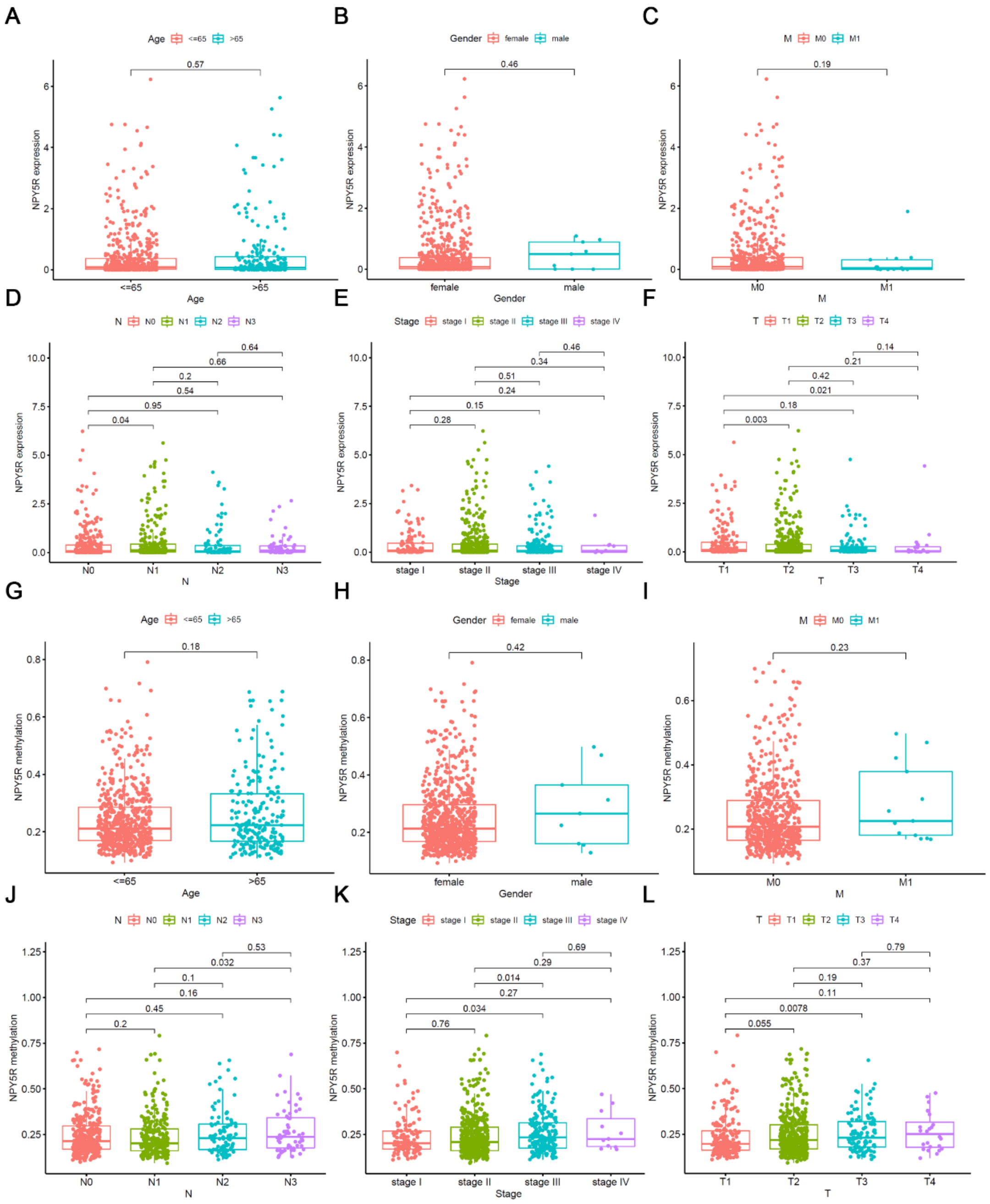

Supplementary Figure 2. The correlation of NPY5R expression and methylation with clinicopathological features of patients with BC. (a–l) The BRCA data set from TCGA was used to analyze the levels of NPY5R expression and methylation based on the clinical parameters of BRCA (age, gender, distant metastasis, lymph node metastasis, TNM stage, and T classification). (a–f) The NPY5R expression was significantly associated with lymph node metastasis and T classification, but not with age, gender, distant metastasis, and TNM stage. (g–l) The methylation level of NPY5R was closely associated with the lymph node metastasis, TNM stage, and T classification, but not with other clinicopathological features including age, gender, or distant metastasis. Statistical significance was evaluated using Wilcoxon rank sum

test.

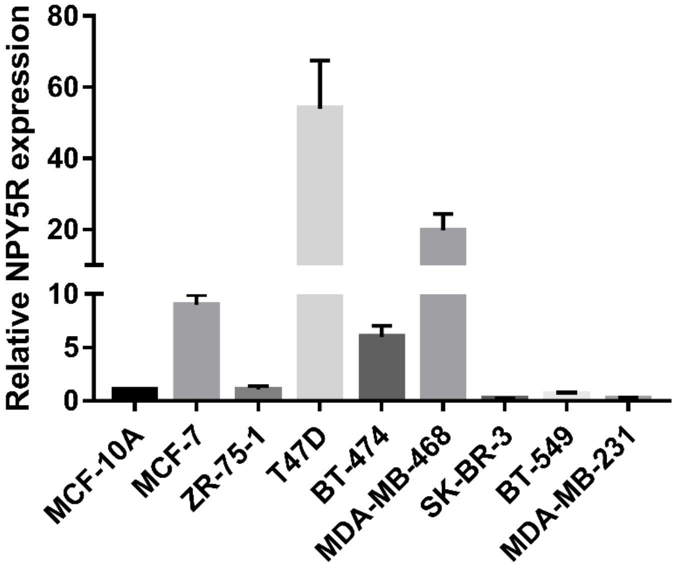

Supplementary Figure 3. qRT-PCR was used to detect NPY5R mRNA levels in normal mammary epithelial cell line and BC cell lines.

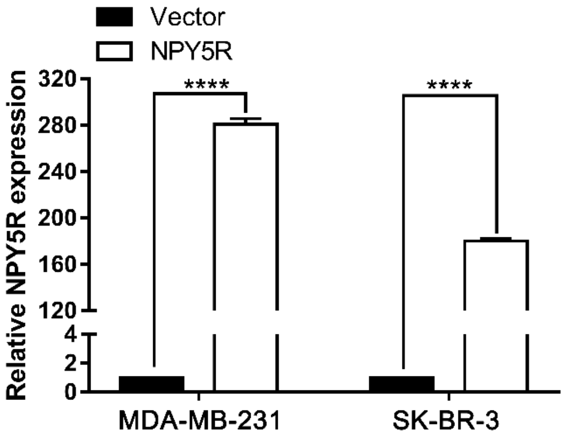

Supplementary Figure 4. qRT-PCR was used to detect NPY5R mRNA levels in NPY5R- and empty vector-transfected cells.
